# Butyrate Protects against Clostridium difficile Infection by Regulating Bile Acid Metabolism

**DOI:** 10.1128/spectrum.04479-22

**Published:** 2023-06-23

**Authors:** Siqi Wang, Leyang Xiang, Fang li, Wenlin Deng, Pinjing lv, Ye Chen

**Affiliations:** a Department of Gastroenterology, The First Affiliated of Guangzhou Medical University, Guangzhou Medical University, Guangzhou, China; b Department of Gastroenterology, Nanfang Hospital, Southern Medical University, Guangzhou, China; c Department of Hepatobiliary Surgery, The First Affiliated Hospital of Jinan University, Guangzhou, China; d Department of Gastroenterology, Hainan General Hospital, Hainan Affiliated Hospital of Hainan Medical University, Haikou, China; e Department of Gastroenterology, Integrative Clinical Microecology Center, Shenzhen Hospital, Southern Medical University, Shenzhen, China; Shandong First Medical University

**Keywords:** *Clostridium difficile* infection, butyrate, bile acid metabolism, intestinal barrier function, inflammation

## Abstract

Clostridium difficile infection (CDI) is caused by a prevalent nosocomial enteric pathogen, leading to high morbidity and mortality. CDI recurrence after antibiotic treatment is high; therefore, it is necessary to develop novel therapeutics against this enteric pathogen. Butyrate is used to treat many diseases because it provides energy, has anti-inflammatory properties, and maintains intestinal barrier function. An anti-CDI effect for butyrate has been reported; however, the specific mechanism remains elusive. This study aimed to explore the potential role and mechanism of butyrate in the treatment of CDI. Using a CDI mouse model, we found that butyrate significantly inhibited CDI development by regulating bile acid metabolism. Dysregulation of fecal bile acid was significantly higher, and levels of short-chain fatty acids were significantly lower in patients with CDI than those in controls. In CDI mice, butyrate exhibited a protective role by enhancing barrier protection, exerting anti-inflammatory effects, and regulating bile acid metabolism. Butyrate treatment also regulated the production of bile salt hydrolase (BSH) flora and activated farnesoid X receptor (FXR), and its therapeutic effects were reduced in CDI mice treated with BSH or FXR inhibitors. Thus, butyrate treatment may serve as a novel therapeutic approach for patients with CDI.

**IMPORTANCE** Here, we show that levels of fecal short-chain fatty acids (SCFAs), particularly butyrate, are reduced, and normal colon structure is damaged in patients with CDI compared with those in healthy individuals. Bile acid (BA) metabolic disorder in patients with CDI is characterized by increased primary BA levels and decreased secondary BAs. In mice, butyrate alters BA metabolism in CDI and may play a vital role in CDI treatment by promoting secondary BA metabolism. Lastly, butyrate-mediated therapeutic effects in CDI require FXR. Our findings demonstrate that butyrate treatment significantly decreases the severity of CDI-induced colitis in mice and affects BA metabolism and FXR activation, which provides a potential alternative treatment for CDI.

## INTRODUCTION

Clostridium difficile is a Gram-positive anaerobic bacterium that forms spores, which spread via fecal-oral transmission, and is a common cause of intestinal infection (1). Due to its strong resistance to physical and chemical disinfection methods in hospitals, C. difficile affects mainly inpatients, resulting in nosocomial infections, which are a serious public health problem. Significant morbidity and mortality in patients with C. difficile infection (CDI) were reported for more than 400,000 and 29,000 cases, respectively, annually (2). The C. difficile toxin damages the actin structure of cells, inducing intestinal epithelial cell death, intestinal barrier dysfunction, and a life-threatening inflammatory response (3). The clinical disease spectrum of CDI ranges from mild to moderate nonbloody diarrhea and intestinal discomfort to severe pseudomembranous colitis, toxic megacolon, peritonitis, sepsis, and death (4).

An important biological mechanism of intestinal commensal resistance to CDI is the regulation of the intestinal bile acid (BA) metabolome, which affects the bacterial life cycle (5–9). Although the exact mechanism of intestinal microbiota resistance is unknown, it has been attributed to the presence of secondary BAs. *In vitro*, cholic acid (CA) and taurocholic acid (TCA) increase spore germination and growth of C. difficile, while chenodeoxycholic acid (CDCA) inhibits this process. Numerous microbe-derived secondary BAs, such as deoxycholic acid (DCA), lithocholic acid (LCA), and ursodeoxycholic acid (UDCA), resist spore germination and inhibit the growth of vegetative C. difficile and toxin activity (8). *In vivo*, fecal BAs in patients with recurrent and prolonged CDI are characterized mainly by high levels of primary BAs and few secondary BAs (10). CDI is predominantly characterized by neutrophilic inflammation within the colonic mucosa and lumen (11). Neutrophils expressing the chemokine receptors CXCL1, CXCL2, and CCL2 migrate to the colon in response to inflammatory stimuli during CDI (12, 13). Granulocyte colony-stimulating factor (G-CSF) and granulocyte-macrophage colony-stimulating factor (GM-CSF) are essential for the expansion and terminal differentiation of neutrophil progenitor cells (14). Lastly, interferon gamma (IFN-γ) is produced by neutrophils in CDI (15).

Short-chain fatty acids (SCFAs) promote the proliferation of colon epithelial cells in normal intestinal tissue, improve intestinal barrier function, exert immunomodulatory effects by inhibiting nuclear factor-kappa B, and ameliorate inflammation and infection. The number of SCFA-producing bacteria in patients with CDI is significantly lower than that in healthy controls (16). In addition, the intestinal concentrations of SCFAs in CDI-susceptible mice are lower than those in CDI-resistant mice (7). While antibiotic use is a major risk factor for CDI, unfortunately, treatment involves further antibiotic therapy.

Currently, the most efficacious approach for CDI prevention is to limit inappropriate antibiotic use. Fecal microbiota transplantation (FMT) is an effective method for treating recurrent CDI; however, ethical and potential infection risks should be considered. Despite aggressive medical and surgical interventions, current medical technology remains challenging due to the high mortality rate of patients with severe CDI (17, 18). Therefore, safer and more effective treatments are urgently needed worldwide. Butyrate exerts anti-inflammatory effects and maintains intestinal barrier function. Furthermore, potential anti-CDI effects of butyrate have been reported; however, the specific mechanism remains unknown. This study investigated the potential effect and mechanism of butyrate in CDI treatment.

## RESULTS

### BA metabolic profiles in patients with CDI and healthy controls.

**FIG 1:**
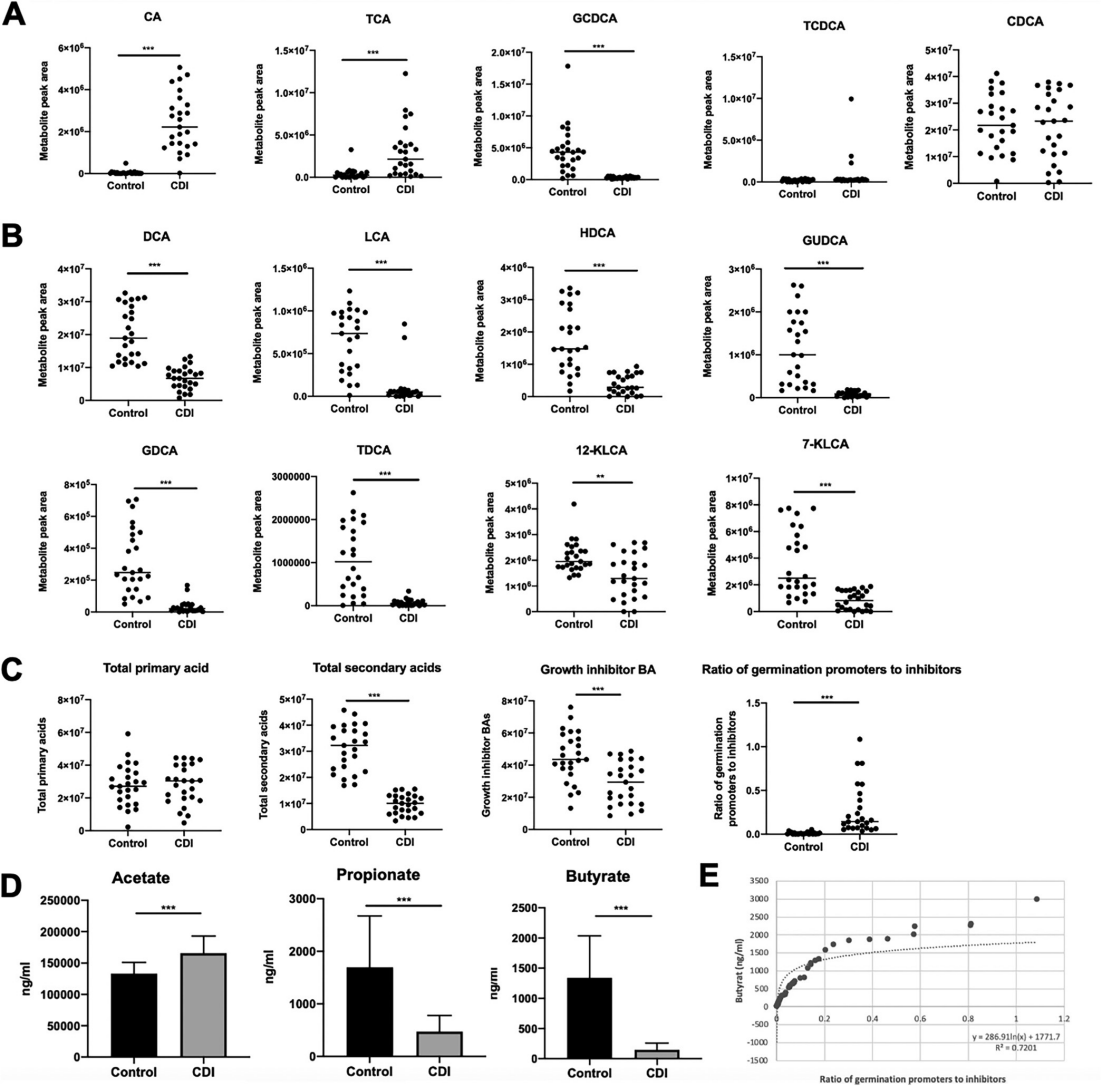
Levels of bile acids (BAs) and short-chain fatty acids (SCFAs) are decreased in fecal samples from human participants. (A) Content of primary BAs. (B) Secondary BA profiles. (C) BA analysis in fecal samples of the healthy control group and patients with CDI. (D) SCFA content. (E) Scatterplots of butyrate concentration and ratio of germination promoters to inhibitors. The *r* values indicate the Spearman correlation coefficients, and *P*-values indicate the statistical significance. Data are shown as mean ± standard deviation (A–C). **, *P* < 0.01; ***, *P* < 0.001.

To understand the aberrant BA metabolism in patients with CDI, we compared different BAs between patients with CDI and healthy controls. The following six primary BAs were identified by liquid chromatography-tandem mass spectrometry (LC-MS/MS): CA, CDCA, TCA, glycochenodeoxycholic acid (GCDCA), taurochenodeoxycholic acid (TCDCA), and glycine combined glycochenodeoxycholic acid (GCDCA). The primary BA contents of CA, TCA, and GCDCA (*P* < 0.001) in CDI patients were significantly higher than those in healthy controls (Fig. 1A).

Eight secondary BAs were detected, as follows: DCA, LCA, glycodeoxycholic acid (GDCA), taurodeoxycholic acid (TDCA), glycoursodeoxycholic acid (GUDCA), hyodeoxycholic acid (HDCA), 7-ketone lithocholic acid (7-KLCA), and 12-ketone lithocholic acid (12-KLCA) (Fig. 1B). All kinds of secondary bile acid levels were lower in patients with CDI than those in healthy controls (*P* < 0.01).

Based on the dramatic differences in BAs caused by C. difficile, we further analyzed the BA profiles. However, there were no significant differences in the total primary levels between healthy controls and patients with CDI (Fig. 1C). Total secondary BA levels and growth inhibitor BAs were significantly lower in patients with CDI than those in controls. The ratio of BAs that promote (CA and TCA) to those that inhibit (CDCA, DCA, LCA, and HDCA) C. difficile spore germination was significantly higher in patients with CDI than that in healthy controls (Fig. 1C). Collectively, these results indicate that BA metabolic disorder in patients with CDI is characterized by an increased content of primary BAs and a decreased content of secondary BAs.

### Butyrate content in patients with CDI is significantly lower than that in healthy controls.

To validate the changes in SCFA levels in patients with CDI, we quantified individual SCFAs in the feces of both groups using LC-MS/MS. Compared with those in healthy controls, patients with CDI had lower levels of propionate and butyrate, with significantly reduced butyrate levels (*P* < 0.001) (Fig. 1D).

The relationship between the level of butyrate and the promotion-to-inhibition ratio for spore germination was strongly inversely logarithmically correlated (*P* < 0.001, r^2^ = 0.7201), suggesting that the potential effect of butyrate in C. difficile is mediated by regulating BA metabolism (Fig. 1E). This finding suggests that the levels of fecal SCFAs, particularly butyrate, were notably lower in patients with CDI than those in controls.

### Intestinal structural damage and increase in inflammatory cells in patients with CDI.

**FIG 2:**
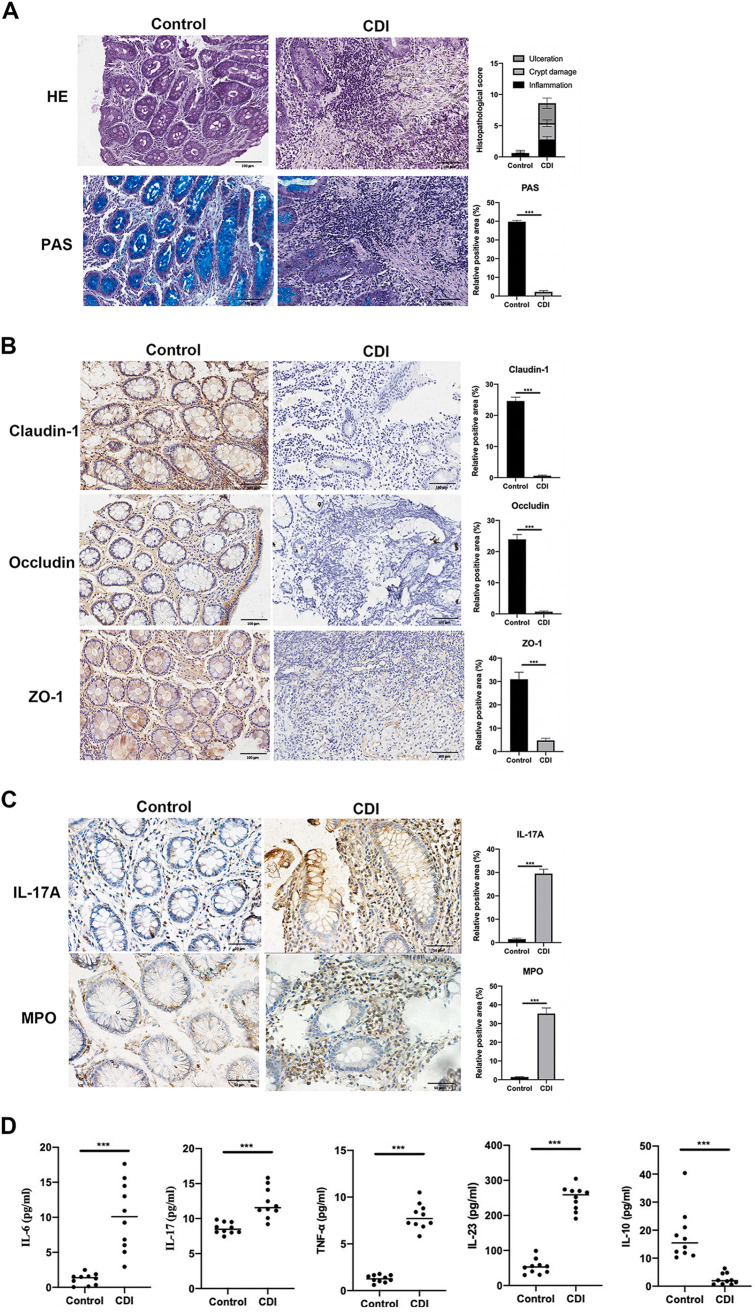
Tissue damage and inflammatory cell infiltration in patients with CDI. (A) H&E and PAS staining of the colon. (B) Representative IHC analysis of tight junction markers Claudin-1, Occludin, and ZO-1. (C) IHC staining of IL-17and MPO in controls and CDI patients tissues. (D) ELISA for the detection of human IL-6, IL-17, TNF-a, IL-23, and IL-10 in serum. ***, *P* < 0.001.

To explore pathological changes in the colon of patients with CDI, the intestinal tissues of healthy controls and patients were observed by hematoxylin and eosin (H&E) and periodic acid-Schiff (PAS) staining. Staining revealed fewer intestinal crypts, a loss of normal glandular structures, an extensive inflammatory cell infiltration, and decreased glycogen in patients with CDI relative to those in controls (Fig. 2A). Furthermore, the expression of the mucosal barrier proteins Claudin-1, Occludin, and zonula occludens 1 (ZO-1) was markedly reduced in patients with CDI compared with those in controls (Fig. 2B), suggesting that severe mucosal impairment occurs in CDI. Moreover, immunohistochemistry (IHC) confirmed that myeloperoxidase (MPO) and interleukin-17 (IL-17) staining were significantly higher in patients with CDI than those in healthy controls (Fig. 2C). Enzyme-linked immunosorbent assay (ELISA) was used to detect the levels of inflammatory factors in the serum. The levels of proinflammatory factors IL-6, IL-17, IL-23, and tumor necrosis factor alpha (TNF-α) in the serum of patients with CDI were significantly higher than those in healthy controls, while the level of the anti-inflammatory factor IL-10 was significantly lower in patients with CDI than that in controls (Fig. 2D). These results suggest that the normal colon structure was damaged in patients with CDI.

### Butyrate improves CDI colitis.

**FIG 3:**
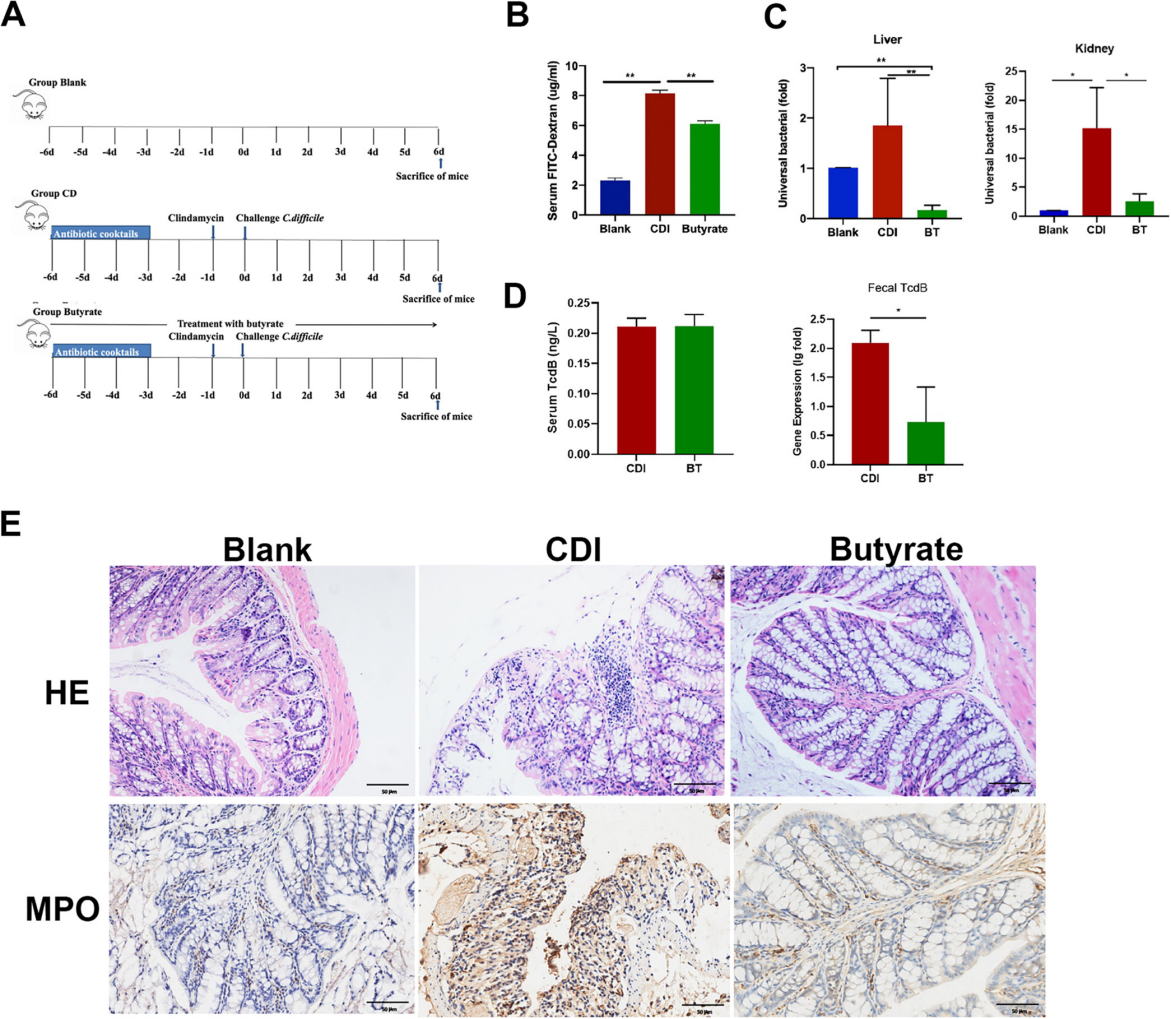
Treatment with 50 mM butyrate alleviates mice colitis. (A) Experimental plan. (B) Serum FITC-dextran. (C) Universal bacterial gene copies in liver and kidney. (D) ELISA analysis of serum TcdB and TcdB mRNA levels in feces. (E) Representative H&E images and IHC staining for MPO. *, *P* < 0.05; **, *P* < 0.01. Scale bar = 50 μm.

To investigate whether butyrate has a direct impact on CDI, three different concentrations of butyrate (10, 50, and 150 mM) were used to treat CDI mice. All diseased mice exhibited weight loss and developed reduced movement, untidy hair, and an arched back. CDI mice started to lose weight after day one, while the butyrate treatment groups experienced a smaller reduction in body weight than the CDI group (see Fig. S1A in the supplemental material). Survival curve analysis showed that on the first day after infection, 10% of mice in the CDI group died, and on the second day, 30% of CDI group mice died. In contrast, the mortality rates in the butyrate treatment groups were significantly lower than those in the CDI group (Fig. S1B). We found that the 50 mM butyrate treatment resulted in the most intact intestinal gland structure without obvious inflammation (Fig. S1C). While tissue damage in the 10 mM butyrate-treated mice was improved, colonic injury persisted in the 150 mM butyrate-treated mice. Therefore, 50 mM butyrate was selected as the concentration for subsequent treatments (Fig. 3A). To explore the side effects of butyrate treatment, 50 mM butyrate was administered orally to untreated healthy control mice for 9 days. Mice administered 50 mM butyrate showed no differences from blank mice, as characterized by similar body weight (see Fig. S2A in the supplemental material), survival (Fig. S2B), and histology (Fig. S2C).

To analyze the permeability of the colon, fluorescein isothiocyanate (FITC)-dextran was administered 4 h before sacrifice at 2 days after infection, and universal bacterial mRNA copies in the liver and kidney were measured. Serum FITC-dextran levels in CDI mice were significantly higher than those in control and butyrate-treated mice (Fig. 3B). In addition, the number of universal bacteria in the liver and kidney was lower in the treatment group than that in the CDI group (Fig. 3C), and feces TcdB gene copies were significant lower in the butyrate-treated CDI mice (BT) group (Fig. 3D).

To better understand the therapeutic effect, we collected intestinal tracts from the mice in different treatment concentration groups and performed H&E staining to evaluate intestinal morphology. During acute CDI, significant tissue damage was observed, as evidenced by epithelial damage and inflammatory cell infiltration (Fig. 3E). In contrast, butyrate-treated CDI mice showed significantly fewer morphological alterations and inflammatory infiltrates than CDI mice. In addition, goblet cell loss due to CDI-induced inflammation was significantly lower in the butyrate-treated mice than that in CDI mice. MPO release upon neutrophil activity in the colon tissue of CDI mice revealed that the number of positively immunostained neutrophils was significantly reduced in the butyrate treatment group compared with that in the CDI mice (Fig. 3E). These results suggest that butyrate treatment may improve CDI effects in mice.

### Butyrate treatment preserves the integrity of the intestinal barrier in mice with CDI.

**FIG 4:**
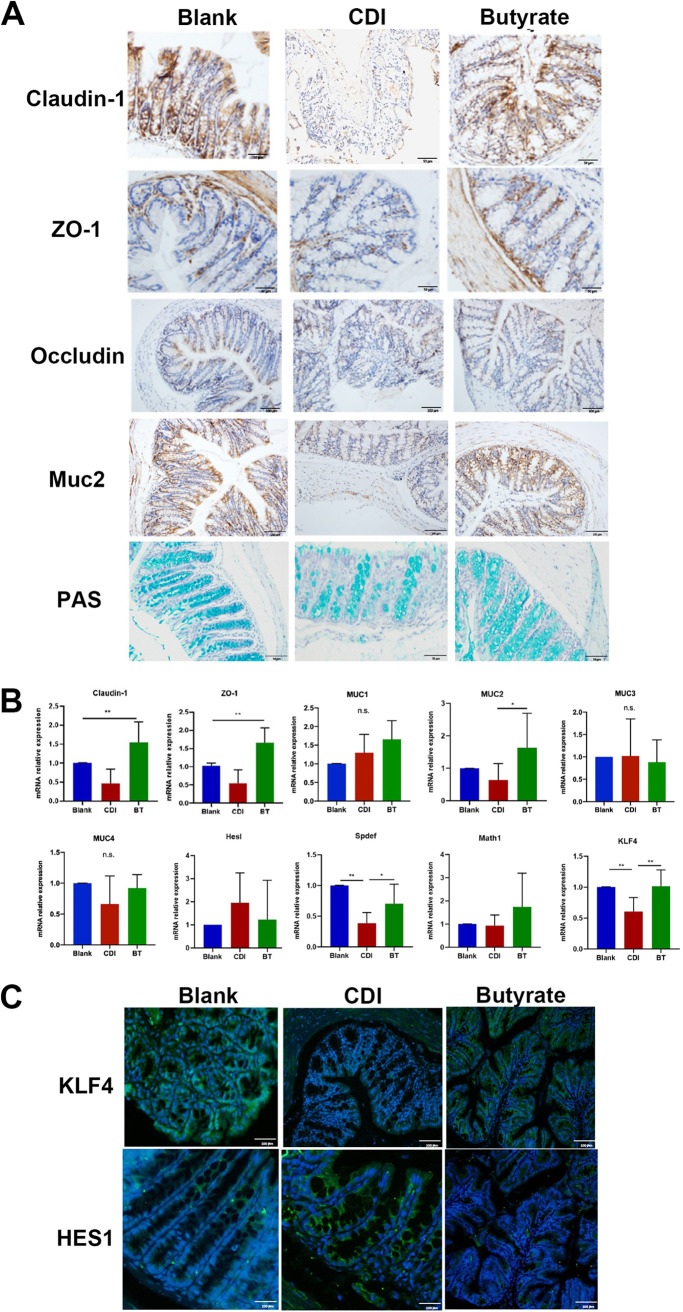
Butyrate facilitates impairment of intestinal barrier function in mice with CDI. (A) Representative IHC analysis of intestinal barrier markers in colonic tissue. (B) RT-PCR analysis of barrier genes in colon. (C) Immunofluorescent (IF) staining of KLF4 and HES1 from mice with CDI and butyrate treatment. *, *P* < 0.05; **, *P* < 0.01; n.s., not significant.

Disruption of the intestinal barrier was observed in CDI mice, and butyrate improved the intestinal barrier after C. difficile attack. Next, we determined the effect of butyrate treatment on barrier improvement. Claudin-1, ZO-1, and Occludin are well-known markers for evaluating tight junction barriers. Claudin-1 was strongly expressed, and ZO-1 and Occludin staining of the intestinal mucosa was visible in the butyrate treatment group relative to that in the CDI group (Fig. 4A). However, after butyrate treatment, Claudin-1 and ZO-1 antigen levels were significantly increased (Fig. 4A), and Claudin-1 and ZO-1 mRNA levels were also visibly increased (Fig. 4B).

The mucus barrier is an important component of the intestinal barrier and is secreted mainly by goblet cells. Secretory mucin MUC2 and nonsecretory mucins MUC1, MUC3, and MUC4 are expressed in the colon. We found that MUC2 and PAS staining was significantly increased after treatment with butyrate compared with that in the CDI group (Fig. 4A), and reverse transcription quantitative-PCR (RT-qPCR) results confirmed this finding (Fig. 4B).

KLF4, HESl, and SAM pointed domain-containing ETS transcription factor (SPDEF) are required for secretory cell differentiation, in contrast to HES1 (19–21). Their expression was assessed using RT-qPCR assays. Mice treated with butyrate showed higher KLF4 and SPDEF expression than CDI mice (Fig. 4B). Furthermore, immunofluorescence assays indicated that butyrate-treated mice showed an increase in KLF4 staining and a decrease in HES1 staining relative to that in CDI mice, suggesting that butyrate increased the number of goblet cells by promoting their differentiation (Fig. 4C).

### Butyrate treatment inhibits inflammatory gene expression and promotes anti-inflammatory gene expression in CDI-induced colitis.

**FIG 5:**
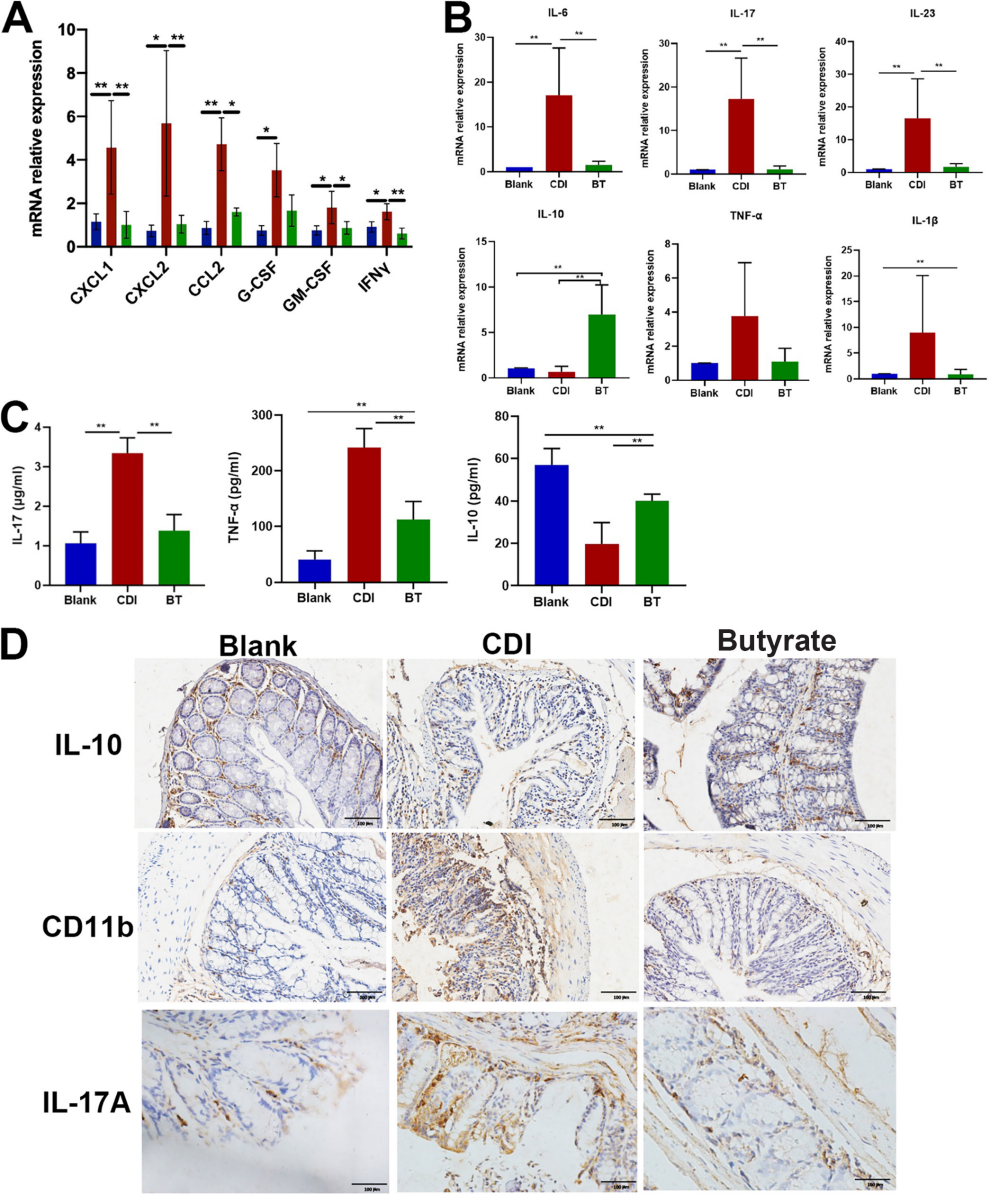
Butyrate decreases immune cells infiltration in CDI mice. (A) mRNA expression of neutrophil marker in colon tissue. (B) RT-PCR analysis of immune markers in colon. (C) Serum ELISA of IL-17, IL-10, and TNF-α production. (D) IL-10, CD11b, and IL-17 staining of colon tissue. *, *P* < 0.05; **, *P* < 0.01. Scale bar = 100 μm.

As CDI is thought to result from both compromised intestinal epithelial barrier function and dysregulation of the mucosal immune system, we investigated whether butyrate modulates inflammatory gene expression in CDI-induced colitis. mRNA expression of CXCL1, CXCL2, CCL2, G-CSF, GM-CSF, and IFN-γ in neutrophils was significantly increased during CDI compared with that in healthy controls, and butyrate treatment reversed this increased expression in the colon (Fig. 5A).

Butyrate-treated CDI mice also showed significantly decreased colonic mRNA expression of the proinflammatory genes IL-6, IL-17, IL-23, TNF-α, and IL-1b and increased IL-10 expression compared with those in untreated CDI mice (Fig. 5B). To further investigate the systemic inflammatory effect of butyrate, we determined serum cytokine levels by ELISA. IL-17 and TNF-α levels significantly increased upon CDI compared with that in healthy controls, and this increase was partially prevented by butyrate treatment (Fig. 5C). In contrast, decreased IL-10 expression in the blood of CDI mice was reversed in butyrate-treated mice (Fig. 5C). In addition, CD11b and IL-17A staining was increased upon C. difficile excessive engraftment compared with that in controls and decreased upon butyrate treatment (Fig. 5D). These data suggest a butyrate-dependent decrease in the recruitment of immune cells to the colon.

Recent studies indicate that robust T helper 17 (Th17) cell aggregates in CDI lead to more severe tissue damage (22, 23). We addressed whether Th17/regulatory T (Treg) cells in the spleen change upon CDI treatment and whether butyrate counteracts such immune cell changes using flow cytometry. CDI induction triggered a significant decrease in splenic Tregs compared with that in controls, and butyrate treatment prevented this disease-associated decrease (see Fig. S3A in the supplemental material). CDI aggravated Th17 cells; however, butyrate treatment fully counteracted this increase and altered the Th17/Treg ratio. Additionally, IL-17 and retinoic acid receptor (RAR)-related orphan receptor gamma mRNA levels were elevated during CDI relative to those in controls, which were rescued by butyrate treatment. The mRNA levels of transforming growth factor beta (TGF-β) and Foxp3 were lower in the CDI than those in the control group, and butyrate treatment increased TGF-β and Foxp3 mRNA levels (Fig. S3B). Therefore, butyrate decreased the expression of several inflammatory genes and promoted the expression of anti-inflammatory genes, most likely contributing to the amelioration of CDI-induced colitis in mice.

### Butyrate alters the fecal BA metabolome.

**FIG 6:**
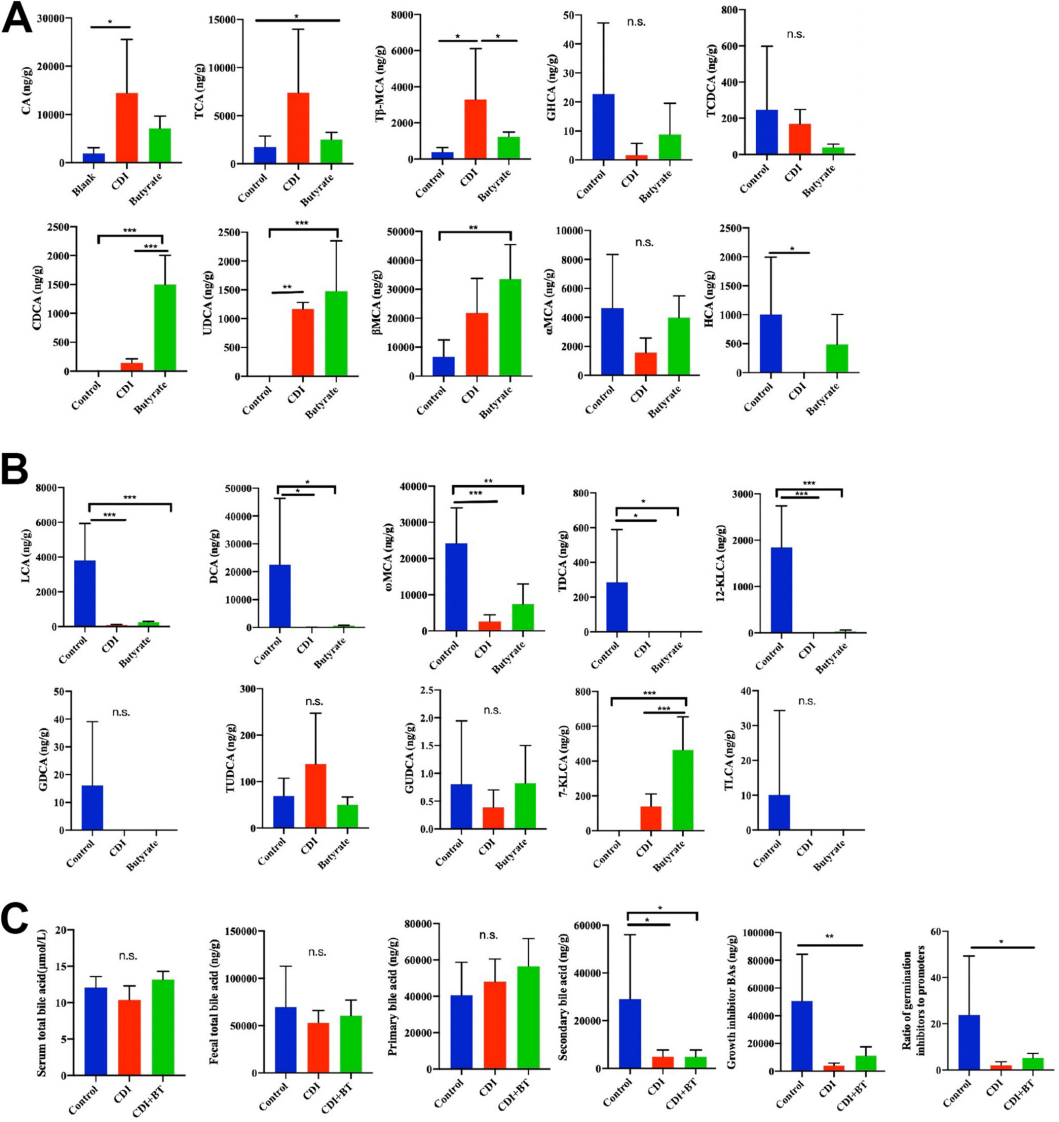
Liquid chromatography-mass spectrometry (LC-MS) detected stool bile acid profiling. (A) Content of primary BAs. (B) Secondary BAs. (C) BA analysis in fecal samples in mice. *, *P* < 0.05; **, *P* < 0.01; ***, *P* < 0.001.

We further explored whether butyrate treatment in CDI mice is an essential pathway involved in regulating BA metabolism. Liver-derived primary BAs and microbiota-derived secondary BAs affect the life cycle of C. difficile (23). Specifically, conjugated primary BAs promote bacterial spore germination, whereas secondary BAs inhibit C. difficile germination and vegetative cell growth. At the end of the experiment, fecal samples were collected to evaluate the effect of butyrate on the gut BA metabolome. Targeted BA analysis via ultraperformance liquid chromatography-mass spectrometry (UPLC-MS) detected 20 BAs in mouse feces. Consistent with the results from human fecal primary BAs, we observed an increase in CA, TCA, and Tβ-MCA in CDI mice compared with that in controls, while butyrate treatment reversed this increase in primary BAs (Fig. 6A). Butyrate treatment was also associated with significantly increased levels of the secondary BAs LCA, DCA, ω-muricholic acid (ωMCA), 12-KLCA, and 7-KLCA in the stool (Fig. 6B).

ELISA analysis of serum BAs revealed that the levels did not significantly differ between groups, and the total fecal BA content and gross primary BA levels in the blank group did not significantly differ from those in the CDI and butyrate groups (Fig. 6C). However, gross secondary BA levels were markedly elevated in the butyrate-treated mice compared with those in the CDI group (Fig. 6C). Categorization of BA metabolites based on their known effects on the C. difficile life cycle revealed that the relative proportion of C. difficile growth inhibitor BAs and the ratio of C. difficile spore inhibitor to germinant BAs were also decreased in CDI mice compared with those in the blank group, while butyrate treatment reversed these BA effects (Fig. 6C). These results indicate that butyrate alters BA metabolism during CDI in mice.

### Butyrate contributes to an increased bacterial abundance of BSH.

**FIG 7:**
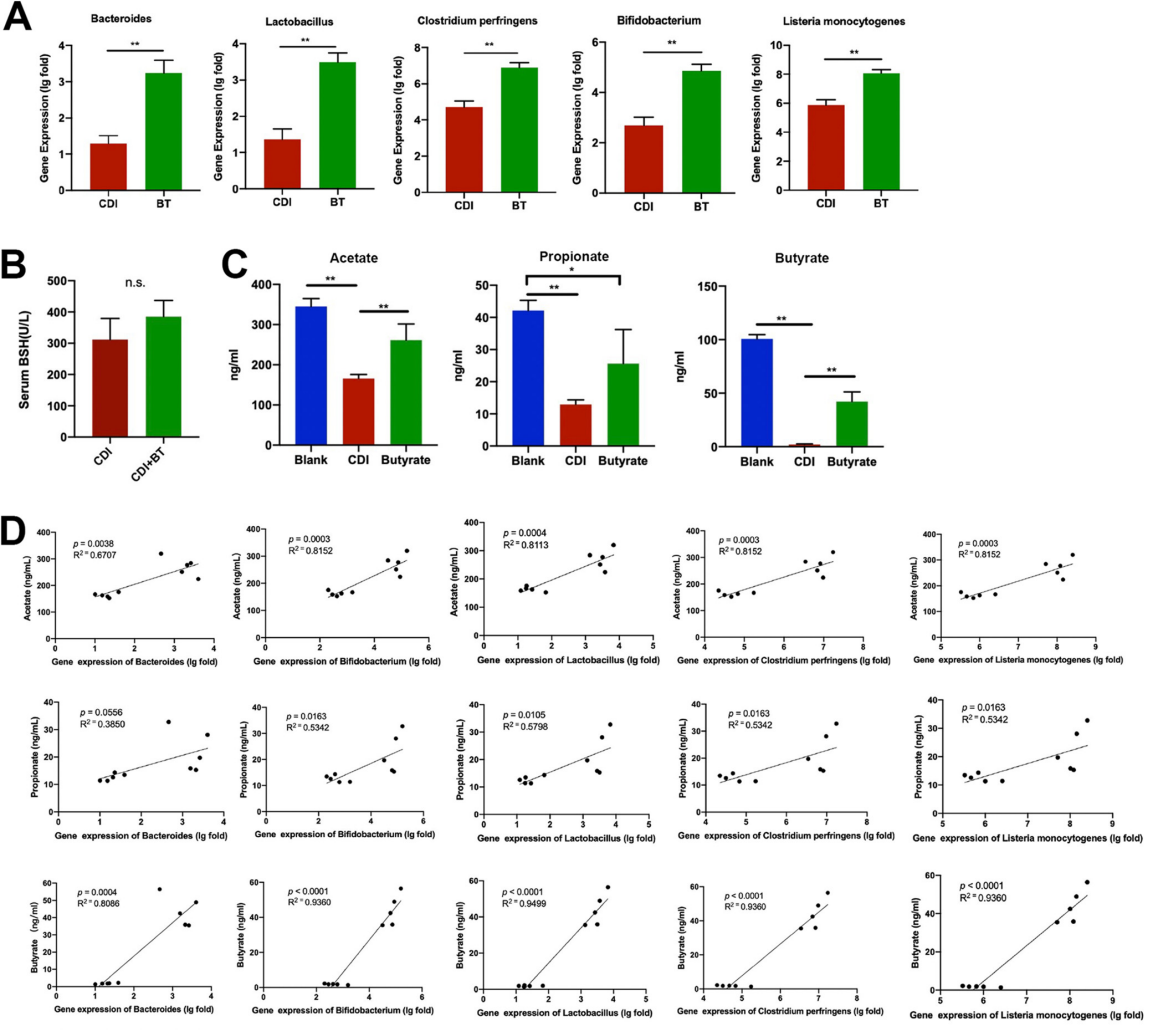
Butyrate treatment improves bacterial abundance of BSH. (A) mRNA expression of BSH-producing bacteria. (B) ELISA of serum BSH. (C) SCFA content analysis. (D) Correlation analysis between BSH-producing bacteria and SCFAs. *, *P* < 0.05; **, *P* < 0.01.

Gut commensals affect colonic BA composition via enzyme-mediated transformation of liver-derived primary BAs. Bacterial-derived BSHs and 7α-dehydroxylases convert conjugated primary BAs into unconjugated and secondary BAs, respectively. BSHs are expressed by bacteria belonging to both *Lachnospiraceae* and *Clostridiaceae* families (24). Further analysis demonstrated that successful butyrate treatment was associated with an enrichment of several bacterial genera, including *Bacteroides*, *Bifidobacterium*, Clostridium perfringens, *Lactobacillus*, and Listeria monocytogenes (Fig. 7A). No significant differences in serum BSH levels were observed between the CDI and CDI + BT groups (Fig. 7B). These results indicate that butyrate treatment of CDI mainly promotes enzymatic reactions by regulating intestinal flora recolonization and recovery of key enzymes and has little effect on serum BSH content.

Next, we quantified SCFA levels in stool samples. All SCFA types were decreased following C. difficile treatment compared with those in controls, and butyrate treatment increased propionate and especially butyrate levels (Fig. 7C). The correlation between bacterial gene copies and SCFA content was further explored using Spearman’s correlation coefficient (Fig. 7D). SCFA levels were positively correlated with levels of BSH bacterial genes, particularly butyrate concentration and BSH bacterial genes, further supporting the conclusion that butyrate regulates BA metabolism to improve CDI colitis. These data indicate that butyrate may play a protective role in bacteria that produce BSH, thereby regulating BAs.

### BSH inhibitors reduce the efficacy of butyrate therapies.

**FIG 8:**
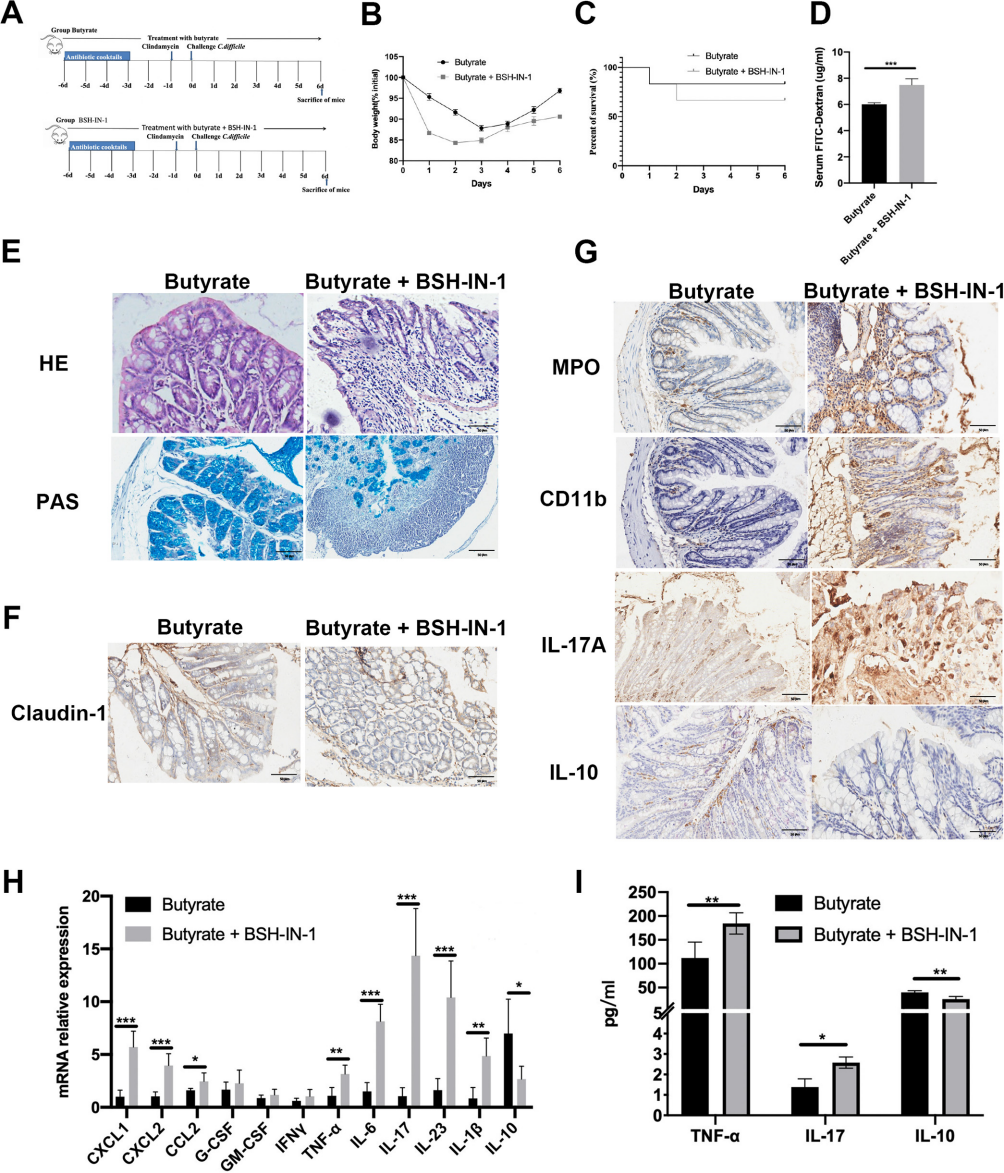
BSH inhibitor reduces the effect of butyrate treatment. (A) Experiment plan. (B) Body weight. (C) Survival. (D) FITC. (E) H&E and PAS staining of colonic sections. (F) IHC staining of Claudin-1 in colon. (G) MPO, CD11b, IL-17, and IL-10 staining in colon. (H) mRNA expression of inflammation factors. (I) ELISA detected TNF-α, IL-17, and IL-10 in serum. *, *P* < 0.05; **, *P* < 0.01; ***, *P* < 0.001. Scale bar = 50 μm.

To confirm whether butyrate improved CDI by regulating BA metabolism, we treated CDI mice with 50 mM butyrate as well as 10 mg/kg of body weight of BSH-IN-1, a BSH antagonist, via gavage twice within a 12-h interval until the end of the experiment (Fig. 8A). BSH-IN-1 treatment significantly decreased body weight to 85% on the second day compared with that following butyrate treatment (Fig. 8B). Moreover, survival decreased by 35% following BSH-IN-1 treatment compared with that in the butyrate mice (Fig. 8C). Serum FITC levels were also higher in the BSH-IN-1 treatment group than those in the butyrate treatment group (Fig. 8D). Consistent with the decrease in body weight and survival, BSH-IN-1 treatment generated significantly more colonic injury, as evidenced by the severity of epithelial damage and inflammatory cell infiltration, than the butyrate-treated mice. PAS staining revealed a loss of goblet cells in the BSH-IN-1 treatment compared with that in the butyrate treatment group (Fig. 8E), mirroring changes in Claudin-1 staining after BSH-IN-1 treatment (Fig. 8F). As expected, BSH-IN-1 treatment significantly restored CDI-induced inflammation. BSH-IN-1 treatment induced CXCL1, CXCL2, CCL2, TNF-α, IL-6, IL-17, IL-23, and IL-1β mRNA expression in the colon and decreased IL-10 expression (Fig. 8H). IHC staining confirmed that severe inflammation occurred with BSH-IN-1 treatment, resulting in elevated MPO, CD11b, and IL-17A staining and decreased IL-10 staining compared with that in the butyrate treatment group (Fig. 8G). To determine the systemic effects of the BSH inhibitor, plasma cytokine production was measured via ELISA. The plasma concentrations of the proinflammatory cytokines TNF-α and IL-17 were elevated following BSH-IN-1 treatment compared with those in the butyrate mice, irrespective of butyrate administration (Fig. 8I). Furthermore, concentrations of the anti-inflammatory cytokine IL-10 were significantly decreased in mice administered BSH-IN-1 compared with that in mice treated with butyrate (Fig. 8I). These findings further indicated that butyrate-mediated BA metabolism protects mice from CDI.

### Butyrate treatment increases intestinal FXR expression in CDI mice.

**FIG 9:**
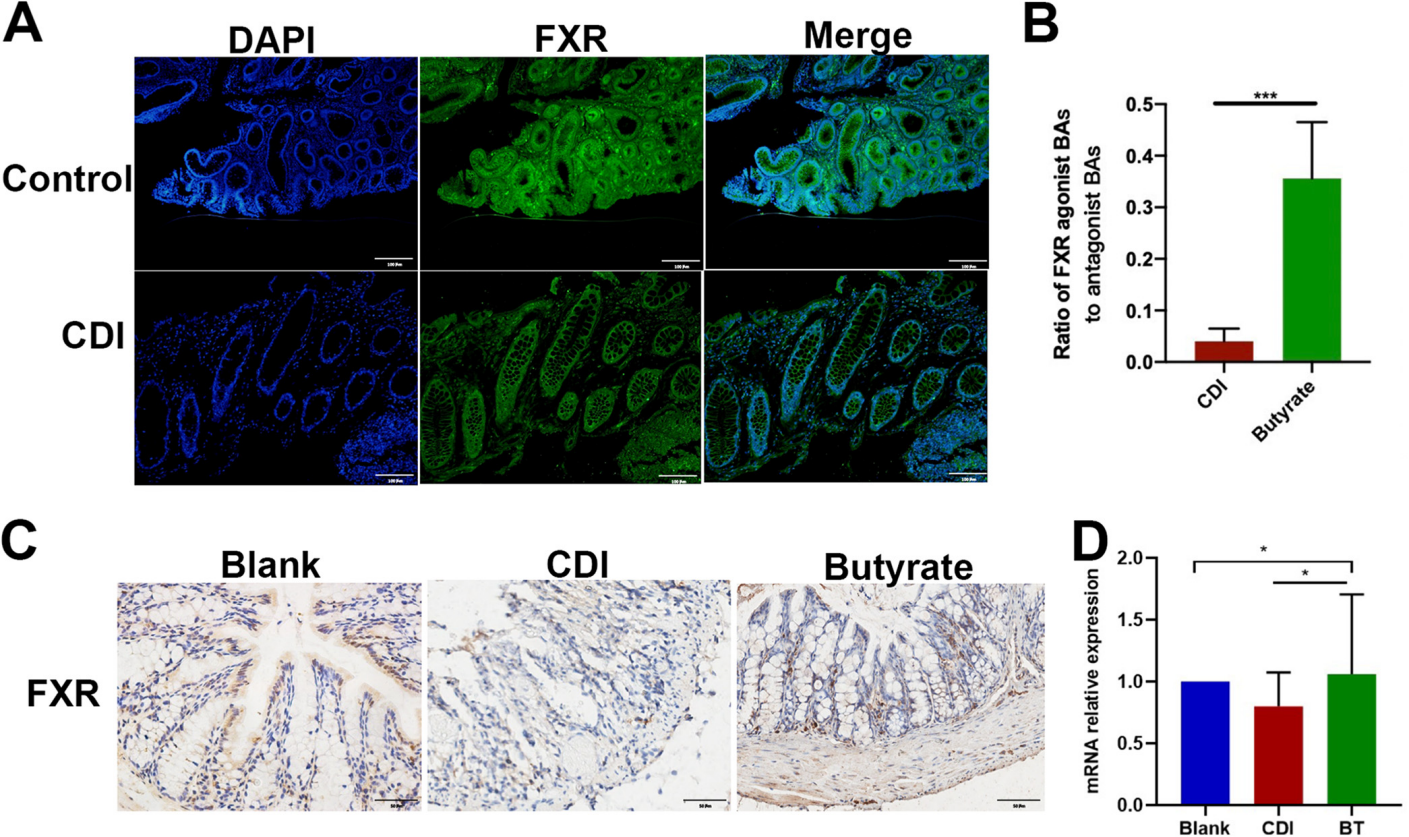
Butyrate treatment activates FXR and ameliorates CDI. (A) Representative IF image of FXR in human colon sections. (B) Ratio of FXR agonist BAs to antagonist BAs. (C) IHC staining of mice colon. (D) mRNA expression of FXR in colon tissue of mice. *, *P* < 0.05; ***, *P* < 0.001. Scale bar = 100 μm for A; scale bar = 50 μm for C.

The bile salt nuclear FXR has been implicated recently in intestinal antibacterial defense and barrier functions. To determine whether FXR correlates with CDI development, we first employed an immunofluorescence assay to detect the expression of FXR in the colon tissues of patients with CDI and paired healthy control tissues. Immunofluorescence staining showed that FXR expression was significantly reduced in patients with CDI (Fig. 9A). However, after butyrate treatment, IHC staining showed that the expression of FXR was significantly increased in CDI mice compared with that in control mice (Fig. 9C), and RT-qPCR showed similar results (Fig. 9D). Many natural BAs activate FXR signaling in a dose-dependent manner (25). Among these BAs, CDCA is the most potent FXR activator, followed by DCA and LCA, whereas CA is the least potent FXR agonist. We further analyzed the ratio of BAs with agonistic (TCA, TCDCA, TDCA, TLCA, GDCA, CA, CDCA, LCA, and DCA) and antagonistic (αMCA, βMCA, ωMCA, and T-βMCA) effects on FXR and found that after butyrate treatment, the ratio of agonistic to antagonistic BAs increased significantly compared with that in controls (Fig. 9B). These results demonstrate that CDI colon tissues exhibit reduced FXR expression levels compared with healthy tissues.

### FXR inhibitors reduce the efficacy of butyrate therapies.

**FIG 10:**
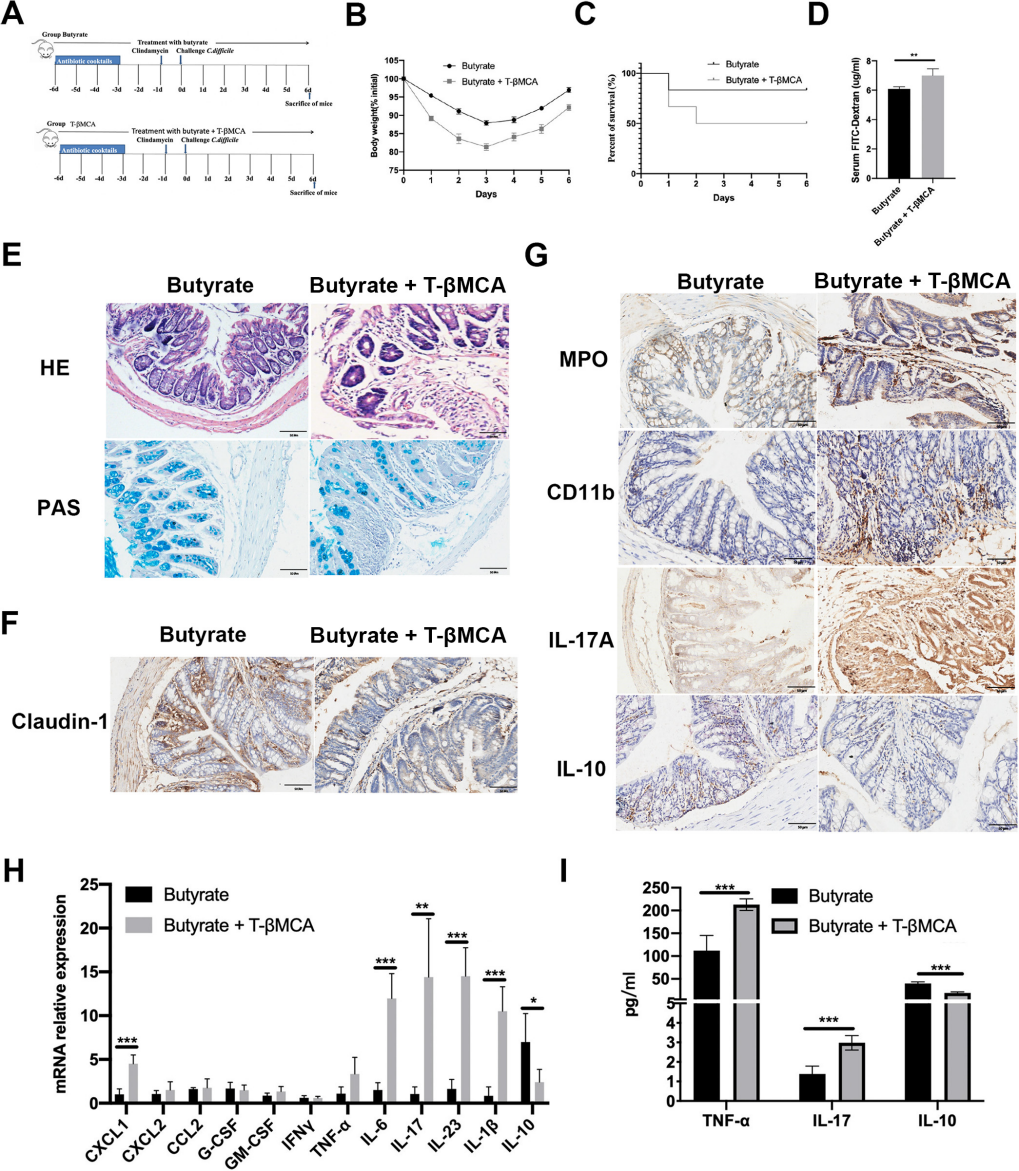
T-βMCA declines the effect of butyrate treatment. (A) Experiment plan. (B) Body weight. (C) Survival. (D) FITC. (E) H&E and PAS staining of colonic sections. (F) The expression of Claudin-1 in butyrate treatment with or without T-bMCA in colon tissue by IHC. (G) The positivity of MPO, CD11b, IL-17, and IL-10 staining in colon samples. (H) RT-PCR analysis of inflammation genes in colon. (I) ELISA analysis of TNF-α, IL-17, and IL-10 levels in serum. *, *P* < 0.05; **, *P* < 0.01; ***, *P* < 0.001. Scale bar 50 μm.

To validate the role of FXR activation, FXR expression was inhibited using T-βMCA (100 mg/kg/day) (Fig. 10A). Daily administration of T-βMCA increased body weight loss (Fig. 10B), whereas survival in the T-βMCA group was lower than that in the butyrate group (Fig. 10C). Serum FITC levels were also higher in T-βMCA-treated mice than those in butyrate-treated CDI mice (Fig. 10D).

H&E staining showed that butyrate treatment of CDI mice resulted in significant epithelial integrity, while the FXR inhibitor partially aggravated the infiltration of inflammatory cells, degeneration of the epithelial layer, and loss of goblet cells (Fig. 10E). The effect of T-βMCA on Claudin-1 decreased upon butyrate treatment (Fig. 10F). Representative IHC images of paraffin-embedded colon sections collected from CDI mice treated with either butyrate or butyrate in combination with T-βMCA revealed elevated MPO, CD11b, and IL-17A staining and decreased IL-10 staining in the T-βMCA group (butyrate + T-βMCA) (Fig. 10G). The efficient suppression effect of T-βMCA on butyrate in the colon was evaluated based on the mRNA expression of inflammation-related cytokines. mRNA expression of proinflammatory cytokines CXCL1, IL-6, IL-17, IL-23, and IL-1b was induced, while the level of anti-inflammatory IL-10 was significantly decreased in T-βMCA-treated mice compared with that in butyrate-treated mice (Fig. 10H). FXR inhibitors alter systemic cytokine concentrations. Absolute concentrations of TNF-α, IL-17, and IL-10 were measured in the plasma of mice treated with either butyrate or butyrate combined with T-βMCA. The amounts of TNF-α and IL-17 detected by ELISA were higher in the blood of mice treated with T-βMCA than those in the group that received butyrate alone, and T-βMCA treatment further reduced IL-10 levels (Fig. 10I). These results indicate that FXR is essential for butyrate-mediated therapeutic effects in CDI.

## DISCUSSION

**FIG 11:**
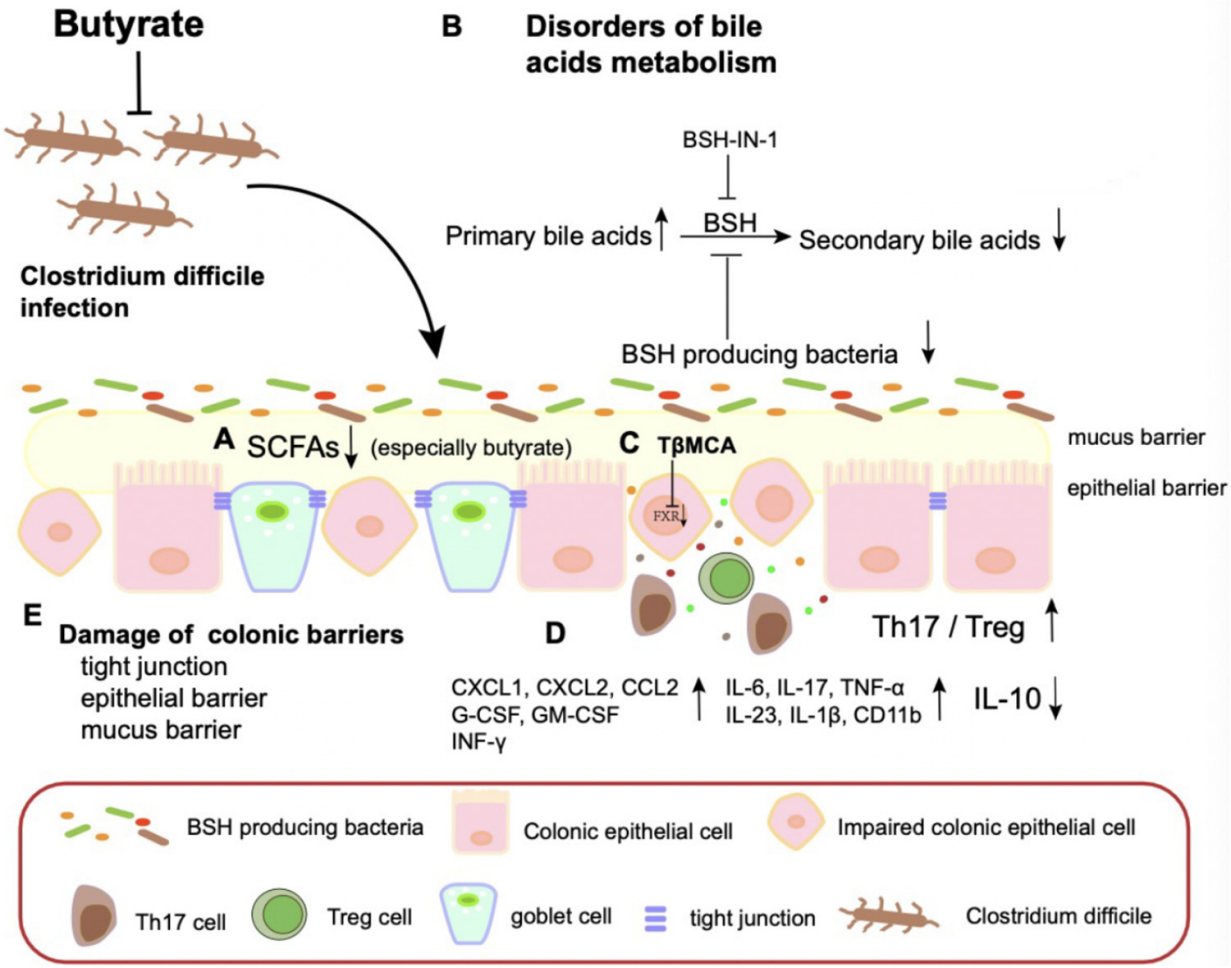
Model of how butyrate attenuates CDI. Butyrate alters disorders of BA metabolism by improving bacterial abundance of BSH to promote secondary BA metabolism, repairs damage colon barrier and alleviates inflammation.

CDI is characterized by the disruption of the intestinal microbiota and barrier, with potentially severe complications and mortality. Current treatment options for patients with CDI consist mainly of antibiotic use to inhibit bacterial growth and FMT to restore intestinal microecology (26). Although current treatments are reasonably effective, significant side effects and treatment failures may occur, highlighting the need for novel treatment options for CDI. Here, we describe butyrate as a potential novel therapy for CDI (Fig. 11).

We used classic murine CDI models, which share many clinical and pathological features with patients with CDI, such as the loss of barrier function and inflammatory response. The intestinal epithelial barrier protects the host by preventing the entry of external antigens and microorganisms into the body. The physical barrier consists of enterocytes, which are tightly connected via intercellular junctions. In addition, goblet cells are intestinal mucin-secreting cells that secrete mucus to form the mucus layer, which protects the mucosal surface from antigens and maintains intestinal barrier function. Patients with CDI display various degrees of epithelial barrier disruption and goblet cell loss, leading to bacterial translocation and inflammation. In our experimental C. difficile colitis model, significant epithelial damage was detected, which was partially prevented by butyrate.

As CDI is thought to result from both dysregulation of the mucosal immune system and compromised intestinal epithelial barrier function in individuals, we explored the ability of butyrate to regulate intestinal inflammation at different levels. Enterocytes serve as immunoeffector cells and secrete cytokines and chemokines to promote inflammation (27). This process results in the infiltration of neutrophils and other immune cells to the site of intestinal inflammation. Neutrophils are absent from healthy human intestinal mucosa. When not properly eliminated, neutrophils contribute to significant tissue damage during acute and chronic diseases (28), suggesting that inhibition of neutrophil migration to the colon may protect against CDI. Intriguingly, levels of intestinal homing chemokines for neutrophils, including CXCL1, decreased with butyrate treatment and increased with BSH and FXR inhibitors compared with those in CDI mice. CXCL1 is a vital factor for recruiting neutrophils in CDI (29, 30). Thus, the protective effect of butyrate against CDI may involve neutrophil depletion and chemokines. However, further experiments are needed to confirm whether butyrate blocks CXCL1 neutrophils. Moreover, we showed that butyrate rescued the depletion of Tregs, prevented an increase in Th17 cells, and increased anti-inflammatory cytokine IL-10 concentration.

FMT is an effective treatment for recurrent CDI (rCDI). However, the mechanism through which FMT affects rCDI remains unclear. Secondary BAs inhibit the vegetative growth of C. difficile, while certain primary BAs promote its germination. In recent years, the recovery of intestinal BA metabolism has become a well-known potential mechanism supported by human and animal studies (8). FMT restores the diversity and composition of intestinal flora and re-establishes BA homeostasis (31). Furthermore, the loss of microbiota-derived bile-metabolizing enzymes may contribute to the pathogenesis of CDI in both mice and humans. Our study showed that butyrate increased the concentration of secondary BAs by regulating the ratio of germination inhibitors to promoters, implying a potential role for butyrate in the regulation of BA metabolism disorders, which may be important in CDI treatment. We further validated this hypothesis using *in vivo* studies. BSH-IN-1 treatment inhibited the effects of BSH, thus inhibiting the production of secondary BAs, which prevented the therapeutic effects of butyrate in CDI mice. These data suggest that butyrate may play a vital role in CDI treatment by promoting secondary BA metabolism.

Nuclear FXR is involved in intestinal immune regulation and barrier function (32). FXR is activated by bile salts and regulates the transcription of genes involved in bile salt synthesis and lipid homeostasis and decreases tumor formation, transport, and metabolism in the liver and intestine by binding FXR responsive elements in the promoters of target genes as a heterodimer with a retinoid X receptor (33). Intestinal biliary salt deficiency is associated with mucosal damage, bacterial overgrowth, and translocation, whereas oral bile salts or FXR agonists counteract these harmful effects (33–37). FXR-null mice exhibit compromised intestinal integrity at baseline, which is not prevented by FXR agonist treatment (36). Moreover, FXR is expressed in activated immune cells and combats the expression of inflammatory cytokines, and regulation of immune cells by FXR may improve intestinal inflammation (38, 39).

In addition to its role in the regulation of metabolism and BA production, FXR signaling plays a role in other CDI-related systemic processes. FXR activation inhibits bacterial overgrowth, blocks ileal mucosal injury in mice (36), and regulates the immune response by reducing inflammatory cytokines (32). Moreover, the inflammatory response or C. difficile itself may reciprocally inhibit the activation of FXR and its target fibroblast growth factor genes, which merits further study (40). Although the effects of FXR on CDI pathogenesis have been examined previously, we comprehensively examined the effect of butyrate on FXR in the regulation of CDI severity. We showed that butyrate treatment increased FXR expression, which may inhibit CDI progression, and validation with T-βMCA treatment favored anti-inflammatory responses in the colon and whole body during CDI. This result demonstrates that amelioration of colitis by butyrate requires FXR.

## MATERIALS AND METHODS

### Ethics approval and consent to participate.

The study involving patients was approved by the Institute Research Medical Ethics Committee of Nanfang Hospital (NFEC-2014-078) and conforms to the Declaration of Helsinki. All patients provided written informed consent to participate in the study.

Additionally, all experiments involving mice were approved by the Animal Ethics Committee of Southern Medical University (K2019013).

### Patient clinical data and sample collection.

CDI diagnosis was based on symptoms, endoscopy, and histological examination, as described previously (41). Patient baseline characteristics, including age, sex, disease severity, and treatment, are shown in Table 1.

**TABLE 1:** Basic information of research objects

Characteristic^*a*^	Data by group		*P* value
	Control (*n* = 25)	CDI (*n* = 25)	
Mean age (yr)	51.76 ± 1.48	51.59 ± 2.03	0.006**
Female (male)	11 (14)	12 (13)	0.30
Ht (cm)	169 ± 0.03	170 ± 0.04	0.08
Wt (kg)	64.36 ± 2.78	63.38 ± 1.76	0.12
BMI	22.53 ± 0.75	22.26 ± 1.00	0.31
Smoking	5	2	>0.99
Symptoms			
Abdominal pain	0	6	0.02*
No. of diarrhea	0	7	0.009**
Pseudomembranous colitis	0	2	0.47
Gastrointestinal hemorrhage	0	0	>0.99
Drug use			
Antibiotic treatment	0	0	0.23
Chemotherapeutics	0	0	>0.99
Immunosuppressive agents	0	0	>0.99
PPI	0	2	0.47
NSAIDs	0	1	>0.99
Diuretics	0	0	>0.99
Cortisols	0	0	>0.99
Prebiotics	0	0	>0.99
Operation treatment	0	0	>0.99
Breathing machine	0	0	>0.99
Dialysis	0	0	>0.99
Gastrointestinal surgery	0	0	>0.99
Organ transplantation	0	0	>0.99
Enema	0	2	0.47
Nasogastric tube	0	0	>0.99
Gastrointestinal endoscope	0	2	0.47
Index
RBC (10^12^/L)	4.83 ± 0.47	4.76 ± 0.40	0.56
WBC (10^12^/L)	6.70 ± 1.90	8.36 ± 3.57	0.42*^b^
Neutrophil (10^9^/L)	3.91 ± 1.29	6.13 ± 3.14	0.002**
ESR (mm/h)	5.41 ± 2.71	9.96 ± 3.75	<0.001***
CRP (mg/L)	1.65 ± 0.96	7.88 ± 5.74	<0.001***
BUN (mg/dL)	4.16 ± 0.83	14.41 ± 3.56	<0.001***
Cr（μmmol/L）	61.64 ± 8.86	60.51 ± 11.74	0.70
Na^+^ (mmol/L)	139.12 ± 2.30	138.99 ± 2.30	0.85
k^+^ (mmol/L)	4.54 ± 0.57	4.25 ± 0.60	0.17
Albumin（g/L）	41.68 ± 4.91	41.43 ± 4.23	0.85
Cholesterol (mmol/L)	4.24 ± 0.92	4.27 ± 0.97	0.92
Triglyceride (mmol/L)	1.02 ± 0.22	1.03 ± 0.32	0.83
HDL (mmol/L)	1.34 ± 0.03	1.26 ± 0.24	0.15
LDL (mmol/L)	2.55 ± 0.44	2.79 ± 0.52	0.32

a BMI, body mass index; PPI, proton pump inhibitor; NSAIDs, nonsteroidal anti-inflammatory drugs; RBC, red blood cells; WBC, white blood cells; ESR, erythrocyte sedimentation rate; CRP, C-reactive protein; BUN, blood urea nitrogen; HDL, high-density lipoproteins; LDL, low-density lipoproteins.

b *, *P* < 0.05; **, *P* < 0.01; ***, *P* < 0.001.

Colon tissue samples were collected from the same gut segment of 25 healthy controls and 25 patients with an initial diagnosis of CDI without any treatment who underwent colonoscopy at the Department of Gastroenterology, Nanfang Hospital, China.

### C. difficile culture.

The C. difficile inoculum was prepared as described previously (42). Briefly, C. difficile strain VPI 10463 (ATCC 43255) was anaerobically grown in cycloserine-cefoxitin-fructose agar (Solarbio, Beijing, China) for 48 h at 37°C, incubated in brain heart infusion liquid medium (Solarbio) for 24 h at 37°C, and then diluted to 1 × 10^8^ CFU/mL for infecting mice.

### Animals.

Male BALB/c mice (6 to 8 weeks old, 18 to 22g) were obtained from the Medical Experimental Animal Center, Southern Medical University, China, and were fed *ad libitum* and housed in a 22 to 24°C temperature- and light-controlled room in a specific-pathogen-free (SPF) laboratory of the Department of Laboratory Animal, Southern Medical University.

Mice were divided randomly into the following six groups after a 7-day adaptation period: control mice (control group), CDI mice (CDI group), butyrate-treated CDI mice (BT group), butyrate-treated mice (control + 50 mM BT group), butyrate and BSH-IN-1 (bile salt hydrolase [BSH] inhibitor)-treated CDI mice (Butyrate + BSH-IN-1group), and butyrate and T-βMCA (farnesoid X receptor [FXR] inhibitor)-treated CDI mice (butyrate + T-βMCA group).

For the butyrate treatment experiment, mice were divided randomly into three groups. The control group received no treatment, and the CDI group mice received an antibiotic mixture in their drinking water containing kanamycin (0.4 mg/mL), gentamicin (0.035 mg/mL), colistin (850 U/mL), metronidazole (0.215 mg/mL), and vancomycin (0.045 mg/mL) for three consecutive days. Two days after antibiotic cessation, clindamycin (20 mg/kg) was administered by intraperitoneal injection, followed by infection with 10^8^ CFU of toxigenic VPI 10463 (ATCC 43255) through oral gavage 1 day later. Butyrate-treated CDI mice (BT group) were gavaged with three different concentrations of butyrate (10 mM, 50 mM, and 150 mM) for 12 days including during CDI modeling.

For the butyrate side effect assay, mice were divided randomly into two groups, as follows: the control group and control + 50 mM BT group.

For BSH function, mice were divided randomly into two groups, as follows: BT group mice were gavaged with 50 mM butyrate and butyrate + BSH-IN-1 group mice were administered BSH-IN-1 by oral gavage (10 mg/kg/day) once a day on the same day as butyrate treatment on CDI mice, which continued until the end of the experiment.

For the FXR inhibitor assay, mice were divided randomly into the following two groups: butyrate-treated CDI mice (BT group) were gavaged with 50 mM butyrate and butyrate + T-βMCA group mice were gavaged with 50 mg/kg/day tauro-β-muricholic acid (T-βMCA), which was synchronized with butyrate treatment on CDI mice for 12 days.

Daily changes in diarrhea, hunchback posture, and wet tail were observed, and weight and survival were monitored during the experiment. At the end of the experiment, mice were euthanized using isoflurane. Tissue samples, including liver and kidney tissues, were harvested for bacterial transplant detection. Colon tissue was used for histopathologic, immunohistochemical, immunofluorescence, Western blotting, and reverse transcription quantitative-PCR (RT-qPCR) analyses. Lastly, cecal contents were collected for bacterial analysis, feces were collected for the toxin B assay, and blood was centrifuged at 3,000 rpm for 10 min to collect supernatant for the inflammatory cytokine assay.

Colon tissues for histology were stored in 4% paraformaldehyde for 24 h and embedded in paraffin. The other tissues were collected immediately, frozen in liquid nitrogen, and stored at −80°C until further analysis.

### Liquid chromatography-tandem mass spectrometry (LC-MS/MS) profiling of fecal BAs.

Fecal samples were thawed on ice. Each sample (100 mg) was homogenized with 300 μL of ice-cold water. Cold steel balls were added to the mixture and then centrifuged for 5 min and incubated on ice for 10 min. Next, the steel balls were removed, 500 μL of pure methanol was added, and then the mixture was centrifuged at 6,000 × *g* for 5 min and incubated on ice for 10 min. The mixture was centrifuged at 13,500 × *g* at 4°C for 10 min, and 600 μL of the supernatant was decanted into another centrifuge tube and concentrated. Next, 100 μL of 5% methanol in water was added to the dried product and centrifuged at 6,000 × *g* for 5 min and then at 13,500 × *g* at 4°C for 10 min. Finally, the supernatant was collected for LC-MS/MS analysis.

All samples were analyzed with the LC-MS/MS system (AB Sciex, MA) following the manufacturer’s instructions. The analytical conditions were as follows: column, Acquity UPLC columns (1.8 μm, 2.1 mm by 100 mm; Waters, Milford, MA; HSS T3 C_18_); column temperature, 35°C; flow rate, 0.3 mL/min; injection volume, 1 μL; solvent system, water (0.01% methanolic acid):acetonitrile; gradient program of positive ion, 95:5 (vol/vol) at 0 min, 79:21 vol/vol at 3 min, 50:50 vol/vol at 5 min, 30:70 vol/vol at 9 min, 5:95 vol/vol at 10 min, and 95:5 vol/vol at 14 min; gradient program of negative ion, 95:5 vol/vol at 0 min, 79:21 vol/vol at 3 min, 50:50 vol/vol at 5 min, 30:70 vol/vol at 9 min, 5:95 vol/vol at 10 min, and 95:5 vol/vol at 14 min.

The original data file obtained by LC-MS/MS analysis were first converted into mzML format using the ProteoWizard software (43). Peak extraction, alignment, and retention time corrections were performed using the XCMS program (44). The support vector regression method was used to correct the peak area. The peaks with a deletion rate of over 50% in each group of samples were filtered. Subsequently, metabolic identification information was obtained by searching the laboratory’s self-built database and integrating the public database with MetDNA.

### Quantitation of SCFAs by GC-MS.

Fecal samples were collected for SCFA analysis using GC-MS as described previously (45). Briefly, the collected cecal contents (200 mg) were mixed with 2 mL ultrapure water and centrifuged at 13,000 rpm for 10 min at 4°C in 4-mL centrifuge tubes. The supernatant was collected; mixed with 50% sulfuric acid (10 μL), 0.5 g sodium sulfate, and 2 mL diethyl ether; and then centrifuged for 10 min at 6,000 rpm. The supernatant was collected, and 50 μL 2-diethylbutyric acid (9.2 mg/mL) was added. The SCFA samples were assessed using a GCMS-Trace1310/ISO LT gas chromatograph (Thermo Fisher Scientific, Waltham, MA).

### Enzyme-linked immunosorbent assay (ELISA).

Cytokine concentrations of tumor necrosis factor alpha (TNF-α), interleukin-17 (IL-17), and IL-10 were measured in mouse plasma, and TNF-α, IL-17, IL-6, IL-23, IL-10, and BSH levels were measured in human serum according to the manufacturer’s instructions (Boswio Biotechnology, Jiangsu, China). Briefly, samples, standard products, and horseradish peroxidase (HRP)-labeled antibodies were added successively to micropores precoated with antibodies, which were then incubated and thoroughly washed. Color was rendered using substrate 3,3′,5,5′-tetramethylbenzidine (TMB), and the concentration of the sample was calculated based on the absorbance (optical density value) of the sample at 450 nm.

### Fluorescein isothiocyanate (FITC)-conjugated dextran assay.

Intestinal permeability was examined using a FITC-conjugated dextran assay. Mice were gavaged with 150 μL FITC-dextran (molecular mass, 4 kDa; 80 mg/mL) (Sigma-Aldrich, St. Louis, MO) for 4 h. Blood was collected and centrifuged at 3,000 rpm for 10 min. Next, the plasma was collected, and FITC concentrations were measured spectrophotometrically in 96-well plates (excitation, 485 nm; emission, 528 nm).

### Histology.

Collected colon tissue samples were fixed in 4% paraformaldehyde (Leagene Biotechnology, Beijing, China) overnight, dehydrated in 70% ethanol, and embedded in paraffin. Sections (4 mm) were stained with hematoxylin and eosin (H&E) according to the manufacturer’s instructions (Leagene Biotechnology).

### Immunohistochemistry (IHC).

Colon tissue sections (4 mm) were deparaffinized in xylene twice for 10 min, rehydrated in a graded ethanol series once for 5 min, incubated in sodium citrate buffer for 10 min for antigen retrieval, blocked with 10% bovine serum albumin for 1 h, and incubated with antibodies against Claudin-1 (1:100; Abcam, Cambridge, UK; ab242370), zonula occludens 1 (ZO-1) (1:100; Abcam; ab276131), Occludin (1:100; Abcam; ab216327), mucin 2 (MUC2) (1:500; Abcam; ab272692), myeloperoxidase (MPO) (1:500; Abcam; ab208670), CD11b (1:100; Abcam; ab133357), IL-17 (2 mg/mL; Abcam; ab79056), IL-10 (10 mg/mL; Abcam; ab189392) overnight, and IL-1β (1:500; Abcam: ab283818). The sections were then incubated with secondary antibodies (SP9000; Zsbio Biotechnology, Beijing, China) for 1 h. The images were acquired using a BX53F microscope (Olympus, Tokyo, Japan).

### Immunofluorescence.

Colon tissue sections were incubated in primary antibodies against Krüppel-like factor 4 (KLF4) (1:50; Thermo Fisher Scientific; 11880-1-AP), HES family basic helix-loop-helix transcription factor 1 (HES1) (1:100; Thermo Fisher Scientific; PA5-28802), and FXR (1:300; Abcam; ab129089) overnight at 4°C. Samples were incubated with secondary antibodies, CoraLite488-conjugated goat anti-rabbit IgG (H+L) (1:500; Proteintech; SA00013-2), for 1 h at 26°C and then incubated with 4′,6-diamidino-2-phenylindole (DAPI) for 3 min. The slides were examined using a BX53F microscope (Olympus) to analyze the expression of KLF4 and HES1.

### Alcian blue-Periodic acid-Schiff staining.

Alcian blue-periodic acid-Schiff (AB-PAS) staining was performed using commercial kits (Solarbio), according to the manufacturer’s instructions.

### RT-PCR.

RNA was isolated from the colon, liver, and kidneys using the TRIzol reagent (TaKaRa Bio, Kusatsu, Shiga, Japan). cDNA was generated from 500 ng of total RNA using the PrimeScript first-strand cDNA synthesis kit (TaKaRa Bio). RT-PCR analysis was performed using the SYBR premix *ex Taq* kit (TaKaRa Bio). The cycle threshold (2^−ΔΔ^*^CT^*) method was used to analyze relative expression levels. Values were normalized to glyceraldehyde 3-phosphate dehydrogenase levels. Primer sequences are listed in Table 2.

**TABLE 2:** Primer sequences for RT-qPCR

Primer name	Sequence	
	Forward	Reverse
Toxin B	TCTGATGCACTATGTGACTTA	ATCTACGAGCGTAGTCAC
GAPDH (mouse)	GGAGAAACCTGCCAAGTATGA	TCCTCAGTGTAGCCCAAGA
Claudin-1 (mouse)	GGTTATCGGAACTGTGGTAGAA	GTGCTCAGGGAAGATGGTAAG
ZO-1 (mouse)	CATCTCCAGTCCCTTACCTTTC	CCTCCAGGCTGACATTAGTTAC
MUC-1 (mouse)	AATCTCATGGTGACAACTCCTT	GGGAGGAACAGAATATGTGAGG
MUC-2 (mouse)	ATGCCCACCTCCTCAAAGAC	GTAGTTTCCGTTGGAACAGTGAA
MUC-3 (mouse)	GCCGTGAATTGTATGAACGGA	CGCAGTTGACCACGTTGACTA
MUC-4 (mouse)	CCTCCTCTTGCTACCTGATGC	GGAACTTGGAGTATCCCTTGTTG
Hesl (mouse)	CCAGCCAGTGTCAACACGA	AATGCCGGGAGCTATCTTTCT
Spdef (mouse)	AAGGCAGCATCAGGAGCAATG	CTGTCAATGACGGGACACTG
Math-1 (mouse)	GAGTGGGCTGAGGTAAAAGAGT	GGTCGGTGCTATCCAGGAG
KLF-4 (mouse)	GTGCCCCGZCTAACCGTTG	GTCGTTGAACTCCTCGGTCT
IL-6 (mouse)	TTTCCTCTGGTCTTCTGGAGTA	CTCTGAAGGACTCTGGCTTTG
IL-17 (mouse)	TTTAACTCCCTTGGCGCAAAA	CTTTCCCTCCGCATTGACAC
IL-23 (mouse)	ATGCTGGATTGCAGAGCAGTA	ACGGGGCACATTATTTTTATCT
IL-10 (mouse)	ACAGCCGGGAAGACAATAAC	CAGCTGGTCCTTTGTTTGAAAG
TNF-α (mouse)	GTAGCCCACGTCGTAGCAAA	ACAAGGTACAACCCATCGGC
IL-1β (mouse)	GAGGACATGAGCACCTTCTTT	GCCTGTAGTGCAGTTGTCTAA
CXCL1 (mouse)	CAAGGCTGGTCCATGCTCC	TGCTATCATTCCTTTCTGTTGC
CXCL2 (mouse)	CCAACCACCAGGCTACAGG	GCGTCACACTCAAGCTCTG
CCL2 (mouse)	GCAGTTAACGCCCCACTCA	CCCAGCCTACTCATTGGGATCA
G-CSF (mouse)	ATGGCTCAACTTTCTGCCCAG	CTGACAGTGACCAGGGGAAC
GM-CSF (mouse)	GGCCTTGGAAGCATGTAGAGG	GGAGAACTCGTTAGAGACGACTT
IFN-γ (mouse)	ATGAACGCTACACACTGCATC	CCATCCTTTTGCCAGTTCCTC
ROR-rt (mouse)	GTGGACTTCGTTTGAGGAAAC	ACTTCCTCTGGTAGCTGGTCAC
TGF-β (mouse)	CTCCCGTGGCTTCTAGTGC	GCCTTAGTTTGGACAGGATCTG
Foxp3 (mouse)	AGGAGCCGCAAGCTAAAAGC	TGCCTTCGTGCCCACTGT
FXR (mouse)	TGAGAACCCACAGCATTTCG	GCGTGGTGATGGTTGAATGTC
16S rRNA	TGCCAGCMGCCGCGGTAA	ACAGCCATGCNCACCT
Universal bacterial	CCGTCAATTCMTTTRAGTTT	CTCTTGAAACTGGGAGACTTGA
*Bacteroides*	CTGAACCAGCCAAGTAGCG	CCGCAAACTTTCACAACTGACTTA
*Bifidobacterium*	CGGGTGAGTAATGCGTGACC	CAGAGACCTGCCTTCGCCAT
Clostridium perfringens	CGCATAACGTTGAAAGATGG	CCTTGGTAGGCCGTTACCC
*Lactobacillus*	AGCAGTAGGGAATCTTCCA	CACCGCTACACATGGAG
Listeria monocytogenes	CGTGCATCGCCCATGTGC	ATCTACGAGCGTAGTCAC

### Flow cytometry analysis.

Flow cytometric analysis of T helper 17 (Th17) and regulatory T (Treg) cells in spleen tissue was performed as described previously (42). Briefly, the spleen was passed through a 200-μm nylon mesh to generate a single-cell suspension and then stained with an FITC anti-mouse CD4 antibody (BioLegend, San Diego, CA; 100406), Alexa Fluor 647 rat anti-mouse IL-17A antibody (BD Biosciences, Franklin Lakes, NJ; 560224), or antigen-presenting cell monoclonal antibody forkhead box P3 (Foxp3; eBioscience, Thermo Fisher Scientific; 17-5773-82). Th17 cells were identified as CD4^+^IL-17A^+^ cells, while Treg cells were identified as CD4^+^Foxp3^+^ cells. Data were analyzed using the FlowJo software, version 7.6 (Treestar Inc., Ashland, OR).

### Statistical analysis.

Statistical analysis was performed using the IBM SPSS Statistics for Windows, version 20.0 software (Armonk, NY) and GraphPad Prism for Windows, version 8.0 (San Diego, CA). Statistical significance was determined using Student’s *t* test or analysis of variance with Bonferroni *post hoc* test, as appropriate. Spearman’s correlation coefficient was used for evaluating the relationships between variables. The results are expressed as the mean ± standard deviation, as indicated in the figure legends. Two-sided *P* values below 0.05 were considered significant.

### Data availability.

The data sets are available from the corresponding author on reasonable request.
