# Supplementary figures and images for: Butyrate Protects against Clostridium difficile Infection by Regulating Bile Acid Metabolism
Source: Microbiol Spectr. 2023 Jun 23;11(4):e04479-22. doi: 10.1128/spectrum.04479-22 (PMC10434071; doi:10.1128/spectrum.04479-22)

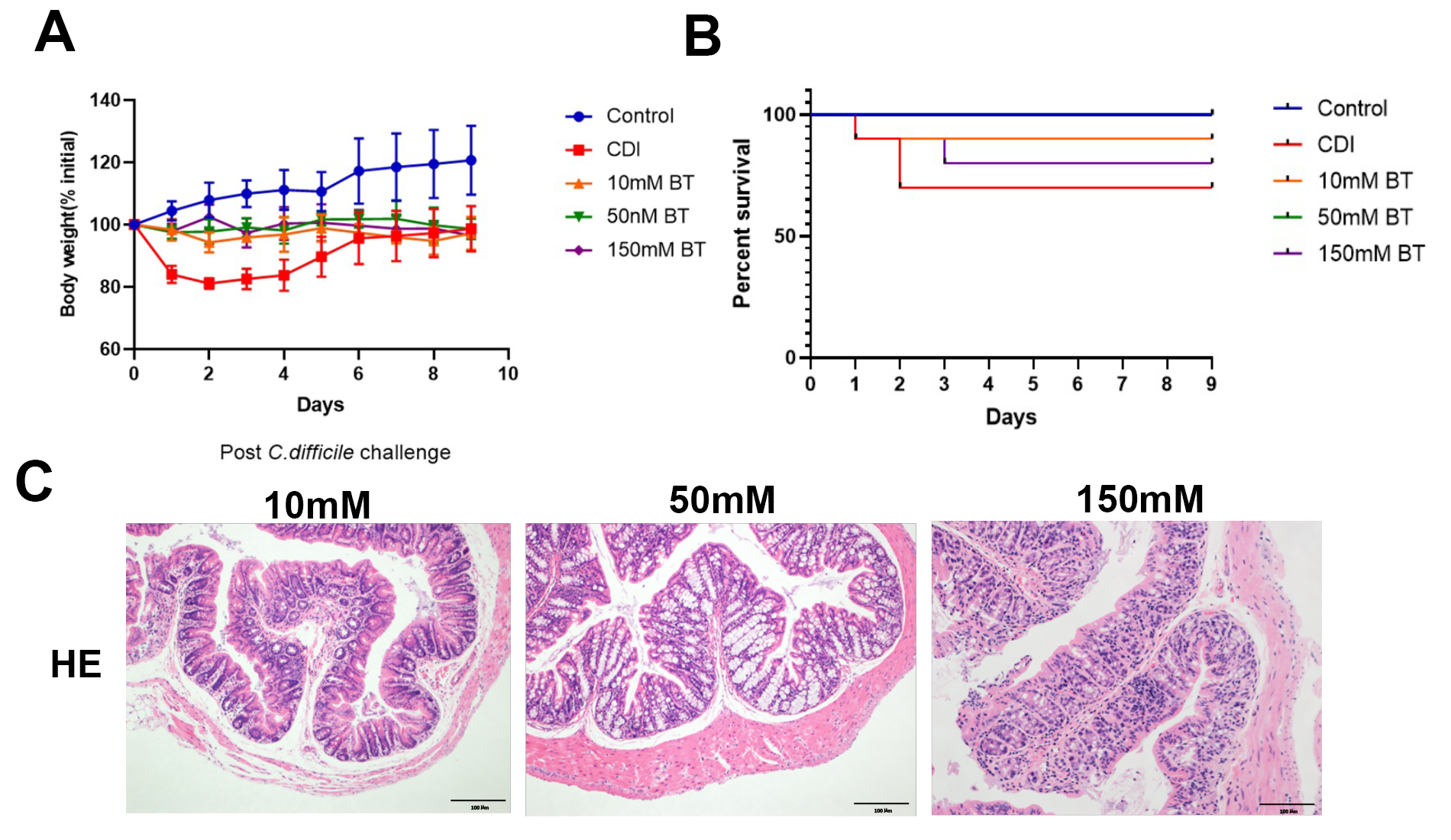

Supplement: Supplemental file 1 — Supplemental material. Download spectrum.04479-22-s0001.tif, TIF file, 8.2 MB [file spectrum.04479-22-s0001.tif]

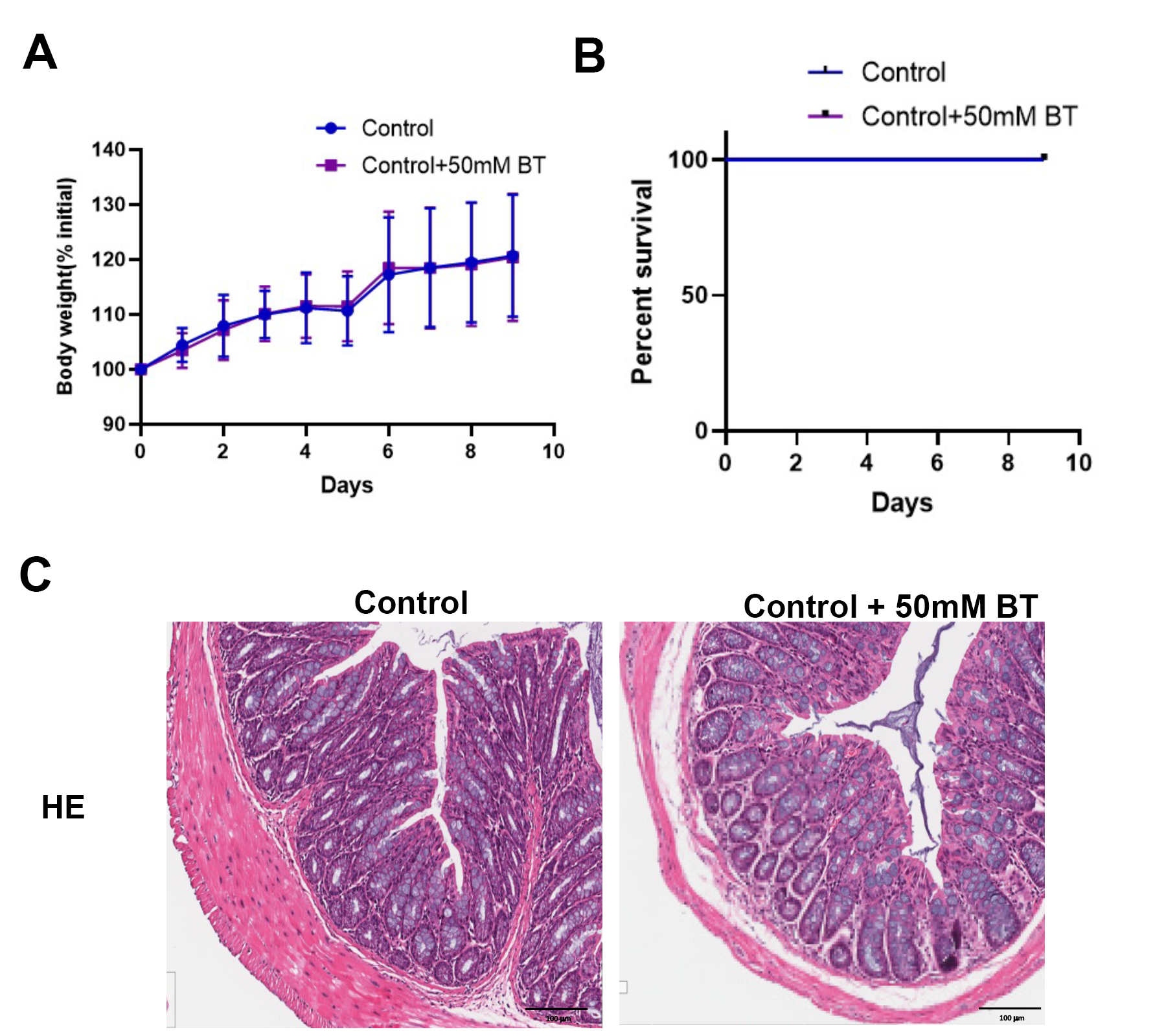

Supplement: Supplemental file 2 — Supplemental material. Download spectrum.04479-22-s0002.tif, TIF file, 11.2 MB [file spectrum.04479-22-s0002.tif]

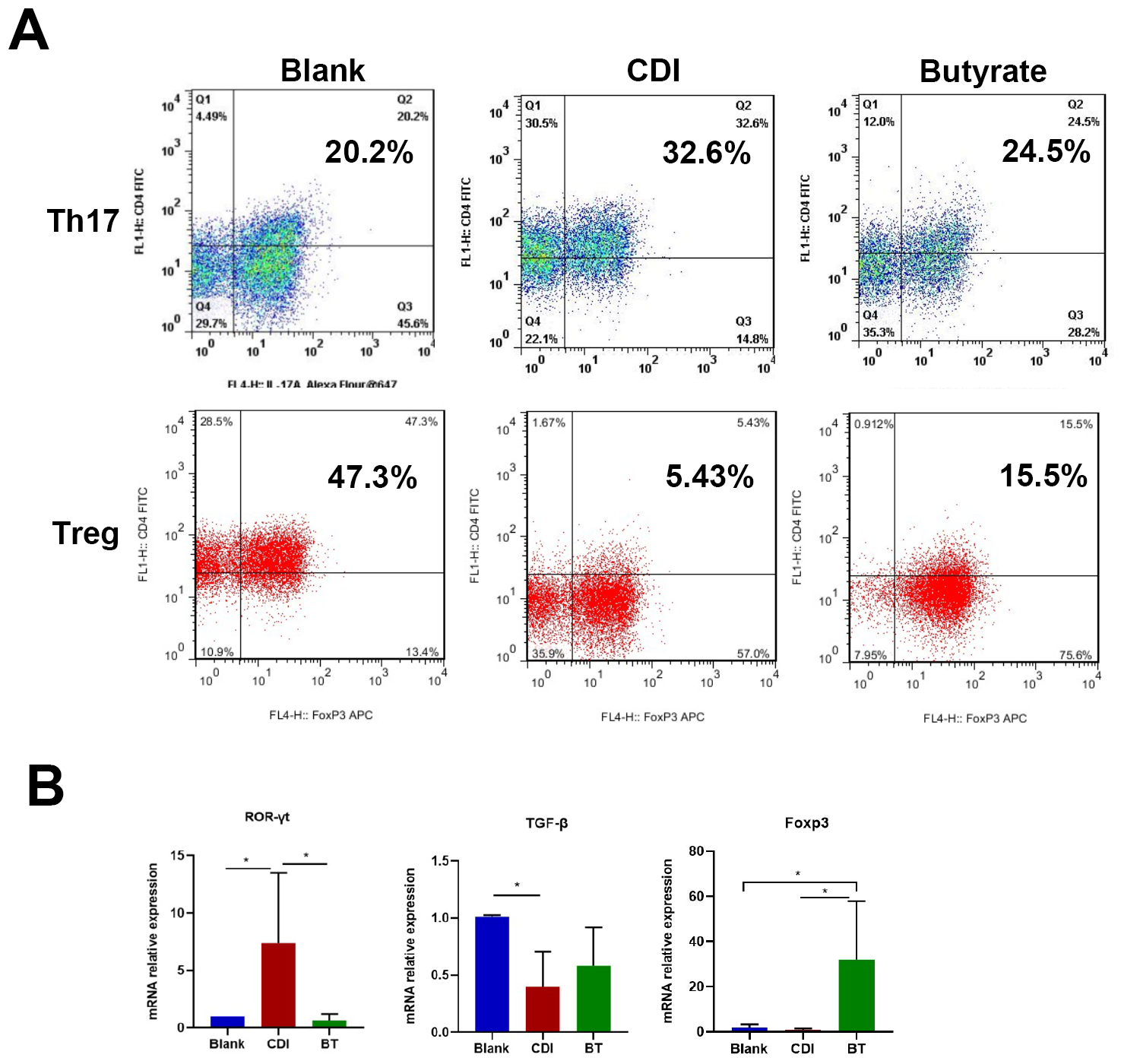

Supplement: Supplemental file 3 — Supplemental material. Download spectrum.04479-22-s0003.tif, TIF file, 7.4 MB [file spectrum.04479-22-s0003.tif]
