# Supplementary material for: Butyrate Protects against Clostridium difficile Infection by Regulating Bile Acid Metabolism
Source: Microbiol Spectr. 2023 Jun 23;11(4):e04479-22. doi: 10.1128/spectrum.04479-22 (PMC10434071; doi:10.1128/spectrum.04479-22)
Supplement: Supplemental file 4 — Supplemental material. Download spectrum.04479-22-s0004.docx, DOCX file, 0.01 MB [file spectrum.04479-22-s0004.docx]

**Supplemental legends**

**Supplement Fig. 1.** Butyrate treatment effects on CDI mice. (A) Body weight change. (B) Survival rate. (C) HE. Scale bar 100 µm.

**Supplement Fig. 2.** 50mM butyrate has no side effects. (A) Body weight change. (B) Survival rate. (C) HE. Scale bar 100 µm.

**Supplement** **Fig. 3.** Butyrate regulates Th17/Treg alters the composition of immune cell populations. (A) FACS analysis of Th17 and Tregs in the spleen from mice. (B)RT-PCR analysis of ROR- γτ, TGF-β and Foxp3 expression.* indicates P < 0.05.
